# MINFLUX fluorescence nanoscopy in biological tissue

**DOI:** 10.1073/pnas.2422020121

**Published:** 2024-12-20

**Authors:** Thea Moosmayer, Kamila A. Kiszka, Volker Westphal, Jasmin K. Pape, Marcel Leutenegger, Heinz Steffens, Seth G. N. Grant, Steffen J. Sahl, Stefan W. Hell

**Affiliations:** ^a^Department of NanoBiophotonics, Max Planck Institute for Multidisciplinary Sciences, Göttingen 37077, Germany; ^b^Georg-August University School of Science, University of Göttingen, Göttingen 37077, Germany; ^c^Centre for Clinical Brain Sciences, The University of Edinburgh, Edinburgh EH16 4SB, United Kingdom; ^d^Department of Optical Nanoscopy, Max Planck Institute for Medical Research, Heidelberg 69120, Germany

**Keywords:** super-resolution microscopy, tissue imaging, postsynapse, 3D nanoscopy

## Abstract

Optical imaging access to nanometer-level protein distributions in intact tissue is a highly sought-after goal, as it would provide visualization in physiologically relevant contexts. Under the unfavorable signal-to-background conditions of increased absorption and scattering of the excitation and fluorescence light in the complex tissue sample, superresolution fluorescence microscopy methods are severely challenged in attaining precise localization of molecules. We reasoned that the typical use of a confocal detection pinhole in MINFLUX nanoscopy, suppressing background and providing optical sectioning, should facilitate the detection and resolution of single fluorophores even amid scattering and optically challenging tissue environments. Here, we investigated the performance of MINFLUX imaging for different synaptic targets and fluorescent labels in tissue sections of the mouse brain. Single fluorophores were localized with a precision of <5 nm at up to 80 µm sample depth. MINFLUX imaging in two color channels allowed to probe PSD95 localization relative to the spine head morphology, while also visualizing presynaptic vesicular glutamate transporter (VGlut) 1 clustering and α-amino-3-hydroxy-5-methyl-4-isoxazolepropionic acid receptor (AMPAR) clustering at the postsynapse. Our two-dimensional (2D) and three-dimensional (3D) two-color MINFLUX results in tissue, with <10 nm 3D fluorophore localization, open up broad avenues to investigate protein distributions on the single-synapse level in fixed and living brain slices.

## Significance

MINFLUX nanoscopy is an emerging optical imaging approach that has closed the resolution gap of fluorescence microscopy for the visualization of biological molecules at the few-nanometer resolution scale. Extending this imaging performance into thick specimens and, in particular, biological tissues is challenged by optical aberrations, and by increased absorption and scattering of the excitation and fluorescence light in the tissue. We demonstrate that the physical hallmarks of MINFLUX—especially its photon efficiency of fluorophore localization, coupled with the confocal background rejection—enable single-digit nanometer resolution at depths of several tens of micrometers in fixed, and ultimately living, brain tissue sections.

The breaking of the diffraction resolution barrier (1) in fluorescence microscopy is an important development in the field of microscopy of cells and tissues. With fluorophore transitions between “on” and “off” states of fluorescence as the central element for feature separation (2), nanoscopy or superresolution (SR) approaches have come in two distinct categories: STED-like (3, 4) coordinate-targeted and PALM/STORM-like (5, 6) coordinate-stochastic SR variants, each with specific strengths and weaknesses.

Aiming to extend the SR capabilities to a range of physiologically relevant contexts, confocal scanning STED imaging has been shown to be applicable deeper down in cells and tissues (7–9), but remains difficult to advance to highest (<20 nm) resolution performance. The wide-field single-molecule-based PALM/STORM methods reach such resolution somewhat more readily, but face challenges inside tissues and at larger imaging depth. The usual implementation of PALM/STORM with total-internal reflection [TIRF, (10)] conditions only allows imaging near the coverslip surface. Therefore, other background-mitigating strategies such as selective plane illumination are effectively required for thicker samples, including tissue applications. In combination with optical sectioning, PALM/STORM was reported to yield 50 to 70 nm resolutions in up to 15 µm imaging depth within living cells and organotypic tissues by employing oil immersion and “digital pinholing” (11), or at 40 to 60 µm depth in fixed tissue slices when utilizing a water immersion lens and angled light-sheet illumination (12). Fluorophore localization by finding the center of the diffraction spot of the fluorescence of individual fluorophores on a camera, as fundamentally used in PALM/STORM, is compromised by background when the fluorophores are located deep inside tissue. A combination of spinning disk confocal microscopy with coordinate stochastic SR by DNA-PAINT was demonstrated in fixed flat cells (13) for two-dimensional (2D) imaging with ~20 nm lateral localization precision in 6.5 µm imaging depth or for three-dimensional (3D) imaging with ~20 nm lateral and ~40 nm axial localization precision in 3 µm imaging depth in the sample.

MINFLUX nanoscopy combines the strengths of the coordinate-targeted and -stochastic approaches. While the fluorescence capability of individual fluorophores is stochastically turned on and off, MINFLUX probes the position of a fluorophore in the sample with a focal excitation pattern containing an intensity minimum, such as a donut-shaped focus. The closer the minimum is to the fluorophore, the fewer fluorescence photons are needed to localize the fluorophore. In a way, the burden of requiring many photons for precise optical localization is shifted from the feeble stream of fluorescence photons to the stable and bright beam of the laser. This fundamental trait of MINFLUX localization brings major advantages as much fewer fluorescence photons are needed to localize a fluorophore with a certain precision. Only the intensity profile of the excitation pattern around the minimum needs to be sufficiently maintained for precise localization, with much less stringent requirements on the detection path. Last but not least, the typical use of a confocal detection pinhole in MINFLUX suppresses background and provides optical sectioning, facilitating the detection of single fluorophores amid a scattering environment such as deeper regions of tissue. Therefore, unlike in PALM/STORM, localization of single fluorophores should be possible deeper down in scattering media. Hence, we decided to investigate whether and to which extent MINFLUX is able to image at the nanoscale deeper inside tissue.

Other approaches to achieve nanoscopy in tissue were reported, such as expansion microscopy (14). As they require harsh chemical alterations of the sample as part of the workflow, their development would never lead to live-cell imaging. In contrast, MINFLUX deserves development because it leaves the sample morphologically largely intact and is inherently compatible with live-cell imaging. Here, we explore this direction. We pioneer MINFLUX imaging in tissue and show that it is possible to obtain resolution, i.e., detail clarity, at the nanometer scale. A major step toward live-tissue nanoscopy, our experiments clarify and address relevant practical challenges and pave the way for its optical feasibility.

Specifically, we describe MINFLUX imaging in tissue sections from the mouse brain, featuring <5 nm localization precisions at up to 80 µm depth, by showing fluorescently labeled Caveolin-1 distributions, and by visualizing actin and PSD95 at the post-synapse at several tens-of-micrometers depth. The potential for live-tissue imaging is exemplified in experiments with PSD95, and MINFLUX in two color channels allowed to probe PSD95 localization relative to the morphology of spine heads. MINFLUX visualizes presynaptic vesicular glutamate transporter (VGlut) 1 clustering and the clustering of α-amino-3-hydroxy-5-methyl-4-isoxazolepropionic acid receptors (AMPAR) at the postsynapse. Extensions to 3D imaging of actin, PSD95, and AMPA receptors demonstrate the potential for investigations of proteins in the volume and at the surface of dendritic spines with remarkable 3D precisions of ~5 nm laterally (*xy*) and 10 to 15 nm axially (*z*) in the complex tissue setting.

## Results

### Adaptations of Experimental Setup.

To date, MINFLUX imaging (15–19) has been demonstrated in relatively flat cells cultured in monolayers, or single immobilized retina layers directly on the coverslip (20). Imaging deeper in tissue comes with a decreased signal-to-background ratio (SBR), because of signal loss due to absorption and scattering in the tissue, an increased background from out-of-focus fluorophores and contributions from tissue autofluorescence. Additionally, for highly precise localizations, the sample position needs to be stabilized against drifts in all imaging depths of interest, which is not possible with the MINFLUX focus lock systems described previously (15, 17). Furthermore, the refractive index mismatch between tissue and immersion oil leads to spherical aberrations, which pose challenges to the control of the focal intensity distribution and the clean formation of its minimum, especially for 3D MINFLUX.

**Fig. 1.**
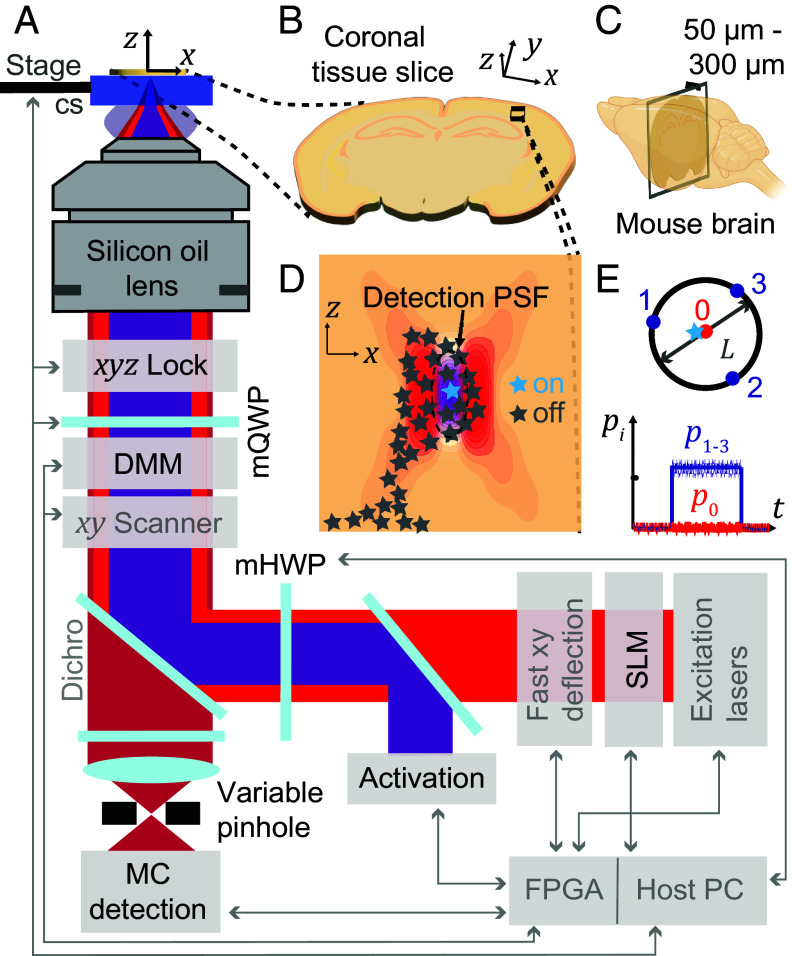
MINFLUX nanoscopy in biological tissue. (*A*) Overview of the custom-built MINFLUX setup for imaging in tissue (adaptations for tissue imaging highlighted in black, standard MINFLUX components shown in gray). (*B*) Sample imaged. The sample is a coronal section prepared by slicing (*C*) a mouse brain into 50-300 micrometer consecutive sections. (*D*) Region of interest in the brain slice showing the optical sectioning capabilities of MINFLUX by the confocal detection volume and the selective on- and off-switching of single emitters. The activated fluorophore is localized photon-efficiently by centering the zero of the donut excitation beam on the activated molecule. (*E*) Targeted-coordinate pattern for the donut excitation beam and schematic photon emission trace of a centered molecule. cs: coverslip; DMM: deformable membrane mirror; mQWP, mHWP: motorized quarter/half-wave plates; *p*_*i*_: photon count ratio in *i*^th^ exposure; MC: multicolor; SLM: spatial light modulator; FPGA: Field-Programmable Gate Array.

The elements of our custom-built beam-scanning MINFLUX system are shown in Fig. 1*A* and more detailed in *SI Appendix*, Fig. S1. Aside from a number of key components required for efficient operation of any MINFLUX nanoscope, such as suitable laser sources, fast beam deflection devices for fluorophore targeting, a fluorescence scanner, and dedicated real-time hard- and software control, several features make our modified implementation more suitable for tissue imaging. Importantly, we chose to use a silicone oil-immersion objective for improved refractive index matching and aberration compensation (compare *SI Appendix*, *Supplementary Text*).

The instrument employs a confocal pinhole with variable size, allowing to adapt to samples of varying fluorophore density and to realize suitable SBR for single-fluorophore detection. Motorized achromatic quarter- and half-wave plates (mQWP/mHWP) control the polarization of the excitation light and enable MINFLUX localizations with a flexible set of different excitation wavelengths (511 nm, 560 nm, and 647 nm) in combination with the photodetectors in a multicolor (MC) detection unit.

A deformable membrane mirror (DMM) as part of the *xyz*-scanning system allows for fast focus shifts and therefore efficient 3D MINFLUX localizations. The DMM can also enable fast aberration correction of the excitation and detection point spread function (PSF) simultaneously. A spatial light modulator (SLM) enables aberration correction of the excitation PSF and imprints the vortex phase mask. There is also an option to provide oxygen for living tissue slices (*SI Appendix*, Fig. S2). Since stability of the sample with respect to the objective lens is critical, we developed an active depth-adaptable focus lock system. Furthermore, we implemented a progressive activation scheme that automates and speeds up the measurement process. The depth-adaptable focus-lock system and the progressive activation are described in detail in the next section.

Samples were usually coronal slices of mouse brain tissue with 50 to 300 µm thickness (Fig. 1*B* and *C*). An imaging region of interest in the brain tissue section can be selected. We chose imaging regions in the cortex or hippocampus. This region of interest was then scanned using a piezo-driven tip-tilt mirror, and sparse fluorophores in the “on” state (Fig. 1*D*) were localized by centering the excitation donut onto them. The centering (MINFLUX localization) was accomplished by iteratively moving the targeted-coordinate pattern (TCP) onto the fluorophore of interest while shrinking the geometric parameter *L* that defines the diameter of the TCP (Fig. 1*E*) (16). In each MINFLUX iteration, the multiplexed emission trace provided a more precise fluorophore coordinate (Fig. 1*E*). If a fluorophore is successfully centered in the TCP, the ratio $p_{0}$ of the number of detected photons $n_{0}$ in the central donut exposure divided by the number of photons *N* collected in all four exposures, should be (close to) zero (compare Fig. 1*E*). All details of the utilized MINFLUX localization sequences can be found in *SI Appendix*, Tables S1–S5.

Both the selective photoactivation of individual emitters in the brain tissue slice and the confocal detection (Fig. 1*D*) contribute to optical sectioning by reducing the out-of-focus background. Selective photoactivation also reduces the bleaching of out-of-focus fluorophores. Filtering of the photon emission traces for emission events during which the fluorophore of interest was centered provides a further means to discard background (*Material and Methods* and *SI Appendix*, Tables S6 and S7).

### Depth-Adaptable Focus Lock System and Progressive Activation.

To allow nanometer-precise localizations, the MINFLUX setup must be stable. This concerns mechanical components (vibrations, thermal drift, stage settling), beam positions (beam jitter due to air turbulence and sound) as well as beam power. The need to actively move the sample with the stage to find the region of interest (ROI) typically resulted in the stage and sample not being equilibrated and, consequently, was a main source of drift during the MINFLUX measurement, which lasted typically tens of minutes for the ROIs in this work. To reduce this drift, active drift correction of the sample with respect to the objective has been employed in MINFLUX imaging (15, 17). The strategy is monitoring and controlling the position of fiducial markers on the coverslip or the coverslip directly, which therefore only works for imaging in close proximity to the coverslip.

**Fig. 2.**
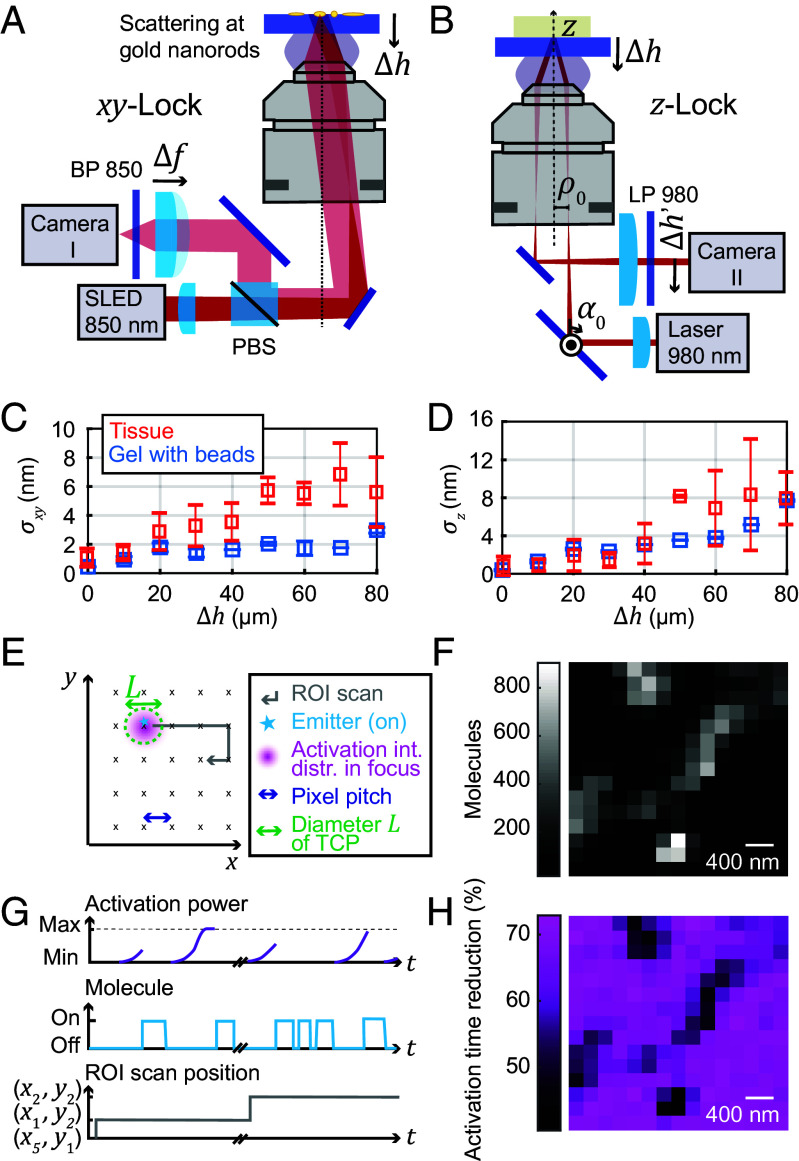
Active drift-correction for depth imaging and progressive activation. (*A*) Depth-adaptable *xy*-lock system. The dark-field images of fiducial markers (gold nanorods) on the coverslip are acquired when imaging in a plane away from the coverslip surface by refocusing the nanorods with a variable-focus lens. (*B*) Depth-adaptable *z*-lock system. The imaging depth range that can be stabilized with high precision is enlarged by a piezo-actuated closed-loop absolute positionable mirror mount. (*C* and *D*) Stability of the (*C*) *xy*-lock position and (*D*) *z*-lock position for measurements in both agarose-sucrose and tissue samples (mean stabilities ± SD). (*E*) Schematic of activation scan over the ROI. (*F*) Number of fluorophores detected at each mosaic scan position. (*G*) Progressive activation, shown schematically. At each new activation location addressed by the mosaic scan, the activation power is ramped up until a molecule starts to emit. (*H*) Reduction in activation time for the progressive activation relative to activation time expected with constant low activation. PBS: polarizing beam splitter, BP, LP: band/long pass, *Δh*: imaging depth, *α*_0_: rotation angle of absolute positionable mirror, *Δh’*: *z*-lock beam position on camera, *σ_xy_*, *σ_z_*: *xy*-lock and *z*-lock position stability, SLED: superluminescent diode.

We constructed a focus lock system that can be adapted to different imaging depths, consisting of two independent parts for stabilizing the sample position perpendicular to the optical axis (the *xy*-lock system, Fig. 2*A*) and for stabilizing the sample position along the optical axis (the *z*-lock system, Fig. 2*B*). For *xy*-position stabilization (Fig. 2*A*), the light from a near-infrared superluminescent LED (~850 nm wavelength) was focused into the back aperture of the objective lens, producing a collimated beam to illuminate an area of about 50 × 50 µm^2^ on the sample coverslip. The gold nanorods applied to the coverslip scattered the light, effecting polarization changes depending on the orientation of the nanorods. While the backreflection from the sample interfaces did not pass back over the D-shaped mirror and was not deflected by the polarizing beam splitter (PBS), the backscattered light from the nanorods did return over the D-shaped mirror and was deflected by the PBS (if its polarization was changed due to nanorod orientation) and then imaged onto a camera by a lens with variable focal length. This variable focus allowed sharp imaging of the nanorods at different axial sample placements. Thereby, active sample stabilization at different imaging depths was achieved.

The position of selected nanorods in the live camera image was fitted by a Gaussian distribution and the position was stabilized by an active feedback loop with a highly precise (0.1 nm positioning resolution) piezo stage. The position stability of the *xy*-lock system was characterized by quantifying the *x*- and *y*-deviations from the mean over time (*SI Appendix*, Fig. S3 *A* and *B*).

The stabilization performance was investigated for measurements in different imaging depths and in different samples. The lock precision in depth in an agarose-sucrose gel (*n* = 1.38; matching the refractive index of mouse brain tissue in layer 1 of the cortex) with embedded fluorescent beads is shown in Fig. 2*C*, along with measurements in different tissue samples for measurement durations of up to 2 h. The focus lock stability (rms uncertainty) $\sigma_{\mathrm{xy}}$ was bounded to <2 nm in the gel sample and ≲6 nm in the tissue at up to 80 µm depth. In practice, fluorophore localization uncertainties below 5 nm could be observed for short measurement times in small fields of view (e.g., caveolin samples; see below).

The stability of the focus lock deep in the sample was independently cross-checked by monitoring the position of fluorescent beads or labeled structures via the main imaging system. Of note, the stability was lower in the tissue sample than in the gel sample, mainly due to scattering of the incoming light in the tissue resulting in a reduced SBR on the camera. The tissue is also less homogeneous than the gel sample, leading to a larger spread in the stability among measurements.

For stabilization along the optical axis (*z*), a separate near-infrared (980 nm) laser beam was focused into the back aperture of the objective lens and impinged collimated on the coverslip–sample interface at oblique incidence (Fig. 2*B*). The reflected light from the coverslip–sample interface passed collimated onto a camera. Using the ray transfer matrix analysis and approximating the objective lens by a thin lens, the position $\Delta h^{\prime }$ of the reflected light on the camera was calculated in a simplified way from the starting parameters $\rho_{0}$ (displacement from optical axis) and $\alpha_{0}$ (angle of incidence) of the incident beam before the objective back aperture, yielding

$$\Delta h^{\prime }=-\frac{2\rho_{0}\Delta hf_{L}}{f^{2}}-\alpha_{0}f_{L}+\frac{2\alpha_{0}\Delta hf_{L}}{f},$$

with the imaging depth Δh, focal length f of the objective lens, and the focal length $f_{L}$ of the lens in front of the camera. For all other parameters kept constant, a movement of the sample along the optical axis directly translated into a movement of the reflected beam on the camera. This effect was used for the active stabilization. A proportional integral (PI) control loop moving the piezo fine stage actively stabilized the center position of the Gaussian intensity distribution on the camera.

There is a trade-off between sensitivity and depth range of the *z*-lock system, because the maximal range of beam movement Δ^*h′*^ was limited by the camera chip size (~4.5 mm). By changing the angle of incidence $\alpha_{0}$ by a piezo mirror, we were able to reliably and precisely switch between a regime with high *z* sensitivity and a regime with larger depth range (*SI Appendix*, Fig. S3*C*). In subsequent measurements, we routinely adjusted the system to the high-sensitivity regime for 0 to 15 µm depth and to a lower-sensitivity regime for 15 to 80 µm depth.

When testing the focus lock system in the *n* = 1.38 gel sample, we documented a slight deterioration of the position stability $\sigma_{z}$ along the optical axis when focusing deeper into the sample, at <4 nm until 60 µm depth and ~8 nm at 80 µm depth (Fig. 2*D*). This was observed to be due to a lower reflectivity of the immersion-oil-to-sample interface when adjusting the *z*-lock for the depth, leading to lower SBR and higher uncertainty of the position. When adjusting $\alpha_{0}$ to the depth, this also led to a slight defocusing of the beam, further decreasing the SBR. We also measured the focus lock precision in tissue samples, observing a similar trend (Fig. 2*D*). At large depth, we noticed lower stability, probably due to reduced SBR caused by the light scattering and absorption of the tissue.

We observed that, when calibrated, and for coverslips of known thickness, the beam position in the *z*-lock system was another means to determine and confirm the imaging depth in the sample besides the position sensors of the coarse and fine stages.

To speed up the MINFLUX acquisitions and to adapt the activation power to inhomogeneous fluorophore densities (Fig. 2*F*), we implemented a progressive activation scheme (Fig. 2 *E* and *G*). The activation power of the 405 nm laser—which served to reactivate both blinking dyes and photoconvert fluorescent proteins—was ramped up exponentially following each detected fluorophore and at each new activation location in the sample. With a low starting activation power, the likelihood of several fluorophores being activated (or photoconverted) at the same time was reduced while maintaining a high fluorophore detection rate due to the about 100-fold ramped-up activation. As the progressive activation procedure acts locally during scanning in a sample-responsive manner, it inherently reduced the time expended in locations where no new fluorophores were activated, whereas it exhausted the fluorophores in crowded locations by activating them successively. To calculate the reduction in activation time due to progressive activation (Fig. 2*F* and *SI Appendix*, Fig. S4), the time needed to activate a molecule with progressive activation was compared to the estimated time needed with constant low activation to reach the same activation dose. Typically, the reduced activation times led to 50 to 75% shorter image acquisition durations.

### MINFLUX Nanoscopy Up to 80 µm Deep in Tissue.

To examine the performance of 2D MINFLUX tissue imaging, we imaged Caveolin-1 labeled with primary and secondary antibody conjugated to the dye Alexa Fluor 647. A well-known blinking buffer for this established dye was used, consisting of an oxygen scavenging system to reduce quenching and photobleaching and a thiol forming a nonfluorescent adduct with Alexa Fluor 647. Caveolin-1 is a membrane protein found in most cells. It is linked to the formation of so-called lipid rafts and the formation of caveolae, which are small (50 to 100 nm diameter), often lightbulb-shaped invaginations of the cell membrane or spherical vesicles in the cell (21–23). Although neurons are one of the few cell types that do not contain typical caveolae structures (24, 25), the Caveolin-1 protein is found in neurons, where it is linked to synaptic plasticity (26).

To investigate differences between MINFLUX imaging in flat cells and complex tissue, we compared Caveolin-1 images and MINFLUX metrics in U-2 OS cells and tissue slices directly on the coverslip (*SI Appendix*, Fig. S5). The median SBR was considerably reduced in tissue (factor >2). Furthermore, the left peak in the histogram of the $p_{0}$ distribution shifted to the right.

We independently checked the emission trace during MINFLUX localization of isolated single molecules on the coverslip surface to quantify and separate different influences on the $p_{0}$ distribution: i) The light intensity in the central minimum of the donut is not strictly zero, but some residual excitation light remains (~0.8% of single-molecule emission is elicited compared to the maximum at the donut crest); ii) There is background from the system, such as dark counts from the avalanche photodiode (APD) detectors, auto-fluorescence, and scattering from the sample, which can be nearly completely suppressed by spectral filtering (a contribution of ~0.7% was determined by single-molecule PSF measurements of the “zero quality” with stepwise bleaching of single fluorophores to the background level; measurement at the glass surface); iii) There is fluorescence from other fluorophores which activate spontaneously outside the targeted volume or do not switch off fully, and whose fluorescence contribution can be only partly suppressed by the confocal detection. This contribution from other fluorophores is deemed to be the most prominent source of background in MINFLUX localizations in a real cell or tissue sample.

**Fig. 3.**
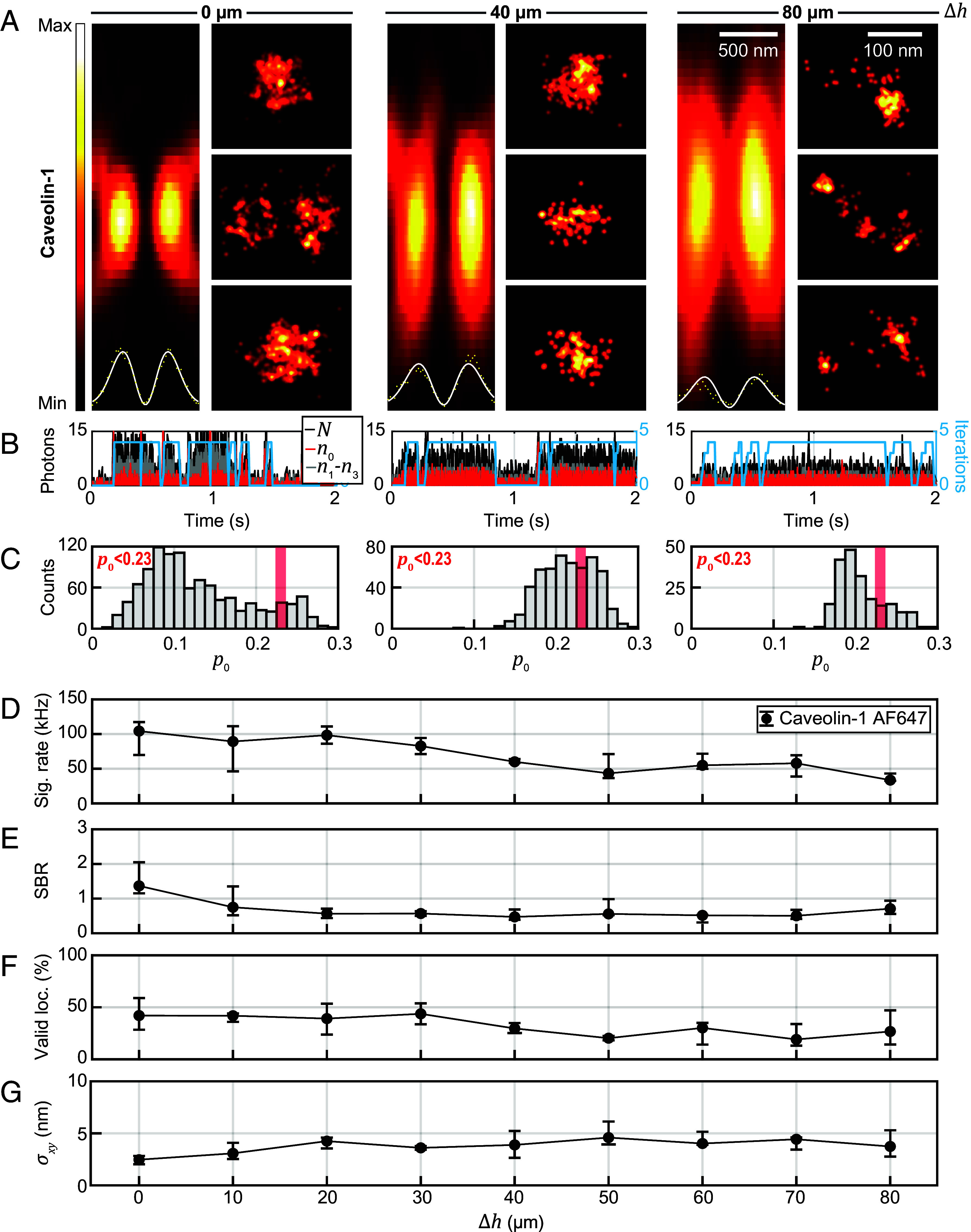
Imaging up to 80 µm deep in tissue with MINFLUX nanoscopy. (*A*) Focal intensity distribution (*xz*) of the excitation donut in different imaging depths in tissue, together with regions of interest from MINFLUX images in the same imaging depth showing Caveolin-1 distributions. Intensity profiles of the excitation light are shown as *Insets* together with a donut-shaped fit along the *x*-axis. The intensity minimum deteriorates from 3% at the coverslip to 14% in 80 µm depth. The FWHM of the focus along *z* is increased from ~900 nm at the coverslip to ~1,660 nm in 80 µm depth. (*B*) Photon traces for the upper row of MINFLUX images. (*C*) Histogram of $p_{0}$ values for resegmented localizations. (*D*) Median signal count rate and interquartile range in each imaging depth. (*E*) Median SBR over depth. (*F*) Median ratio of valid localizations over depth. (*G*) Median localization precision over depth. Statistics from 703,924 localizations (23 images) in ~0 µm, 58,303 locs. (9 images) in ~10 µm, 50,396 locs. (10 images) in ~20 µm, 10,051 locs. (2 images) in ~30 µm, 16,009 locs. (5 images) in ~40 µm, 53,764 locs. (5 images) in ~50 µm, 32,978 locs. (6 images) in 60 µm, 193,465 locs. (15 images) in 70 µm and 12,289 locs. (7 images) in ~80 µm imaging depth. *Δh*: imaging depth, *N*: number of photons collected in one multiplexing cycle. *n_i_*: number of photons in *i*th exposure, *p*_0_ = *n*_0_/*N*, *σ_xy_*: localization precision, SBR: signal to background ratio.

With increasing depth *Δh* from the coverslip, the focal donut pattern became more elongated in the axial direction, and the intensity contrast between minimum and donut crest was reduced, from ~3% at the glass surface to ~14% residual intensity at the center of the pattern at 80 µm depth (Fig. 3 *A* *Insets* in the respective *Left* panels; constant background offset in the image subtracted). The PSFs shown were measured on small Caveolin-1 clusters. A more detailed overview of focal-region donut shapes in different sample depths can be found in *SI Appendix*, Fig. S6 and the accompanying *SI Appendix*, *Supplementary Text*.

The MINFLUX reconstructions (Fig. 3*A*) were found to be comparable to those in previous reports showing fluorescence nanoscopy of caveolae in cells (27–29), similar in morphological appearance, overall dimensions, and the numbers of contained localizations. Single-fluorophore signals above the background level (Fig. 3*B*) showed a decreasing trend with depth (see below), and the histogram of $p_{0}$ values (Fig. 3*C*) exhibited a shift to higher $p_{0}$ with depth, still yielding robust numbers of localizations below the background threshold to enable meaningful reconstructions. From *Δh* = 0 to 80 µm, with increasing depth, the average signal rate decreases from ~100 kcps (kilocounts per second) to ~40 kcps (Fig. 3*D*). The attained SBR, while between 1 and 2 at the surface, was found to plateau at ~0.5 beyond 20 µm depth (Fig. 3*E*), still allowing robust localizations. The ratio of valid localizations according to a $p_{0}$ < 0.23 background filtering criterion decreased by up to 25% over the examined depth range, reaching a plateau at ~25% (Fig. 3*F*). The results reveal that at 40 or 80 µm deep in tissue the background level indeed is higher than at the coverslip surface, where there are only fluorophores in the light cone above the focus position that can contribute to the background, and the signal decreases due to increased absorption and scattering of the excitation and fluorescence by the tissue.

These effects caused a decline in the SBR and the shift of the signal peak in the $p_{0}$ distribution closer to the background peak, making a clear separation based on $p_{0}$ less straightforward with increasing depth. Aberrations introduced by local inhomogeneities in the refractive index and the refractive index mismatch between tissue and immersion oil—even for the more suitable silicone oil system that we utilized—further shifted the signal peak in the $p_{0}$ distribution to the right. The median localization precision decreased with depth, from ~2.5 nm to still <5 nm at Δ*h* = 80 µm (Fig. 3*G*), as was expected for a trend to lower SBR. Robustly attainable precision below 5 nm at several tens of micrometer depth appears remarkable and promising for further investigations. As demonstrated above, part of this experimentally determined precision can likely be attributed to imperfect lateral stabilization, as σ slightly exceeds the Cramér-Rao bound calculated from measured SBR (compare *SI Appendix*, Fig. S7).

### MINFLUX Nanoscopy of Postsynaptic Proteins in Fixed Tissue.

**Fig. 4.**
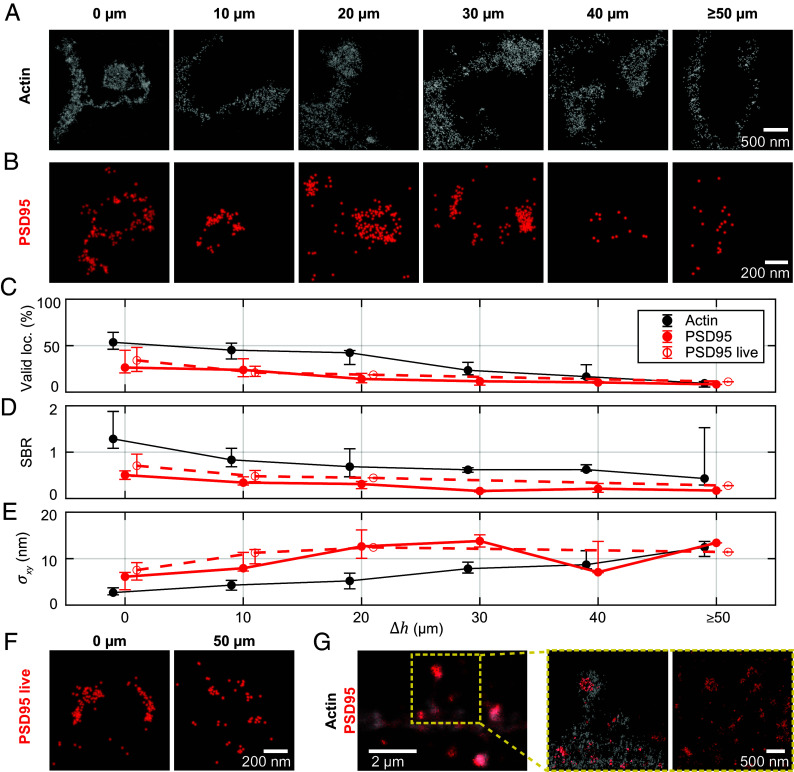
Imaging the post-synapse deep in tissue. (*A*) MINFLUX nanoscopy of actin (LifeAct-EYFP labeled with primary and secondary antibody by Alexa Fluor 647) in 0 to 50 µm imaging depth. Localizations classified by delineation (*SI Appendix*, Fig. S9) as not belonging to spine or dendrite are reduced in visibility. (*B*) PSD95-mEos2 in fixed tissue in 0 to 50 µm imaging depth. (*C*) Median percentage of valid MINFLUX localizations (*p*_0_ < 0.23) and interquartile range over imaging depth, calculated from several images in each imaging depth. (*D*) Median signal to background ratio vs. imaging depth. (*E*) Median localization precision vs. imaging depth. Number of localizations and images from which the statistics for the three different samples are calculated are shown in *SI Appendix*, Table S8. (*F*) PSD95-mEos2 in living tissue in 0 and 50 µm imaging depth. (*G*) Imaging with two excitation colors. Two-color image of PSD95-mEos2 and actin-AlexaFluor647. (*Left to Right*): Two-color confocal overview image. Two-excitation-color MINFLUX in the ROI showing the PSD95 distribution on the postsynapse (only delineated dendritic region shown); full image from the PSD95-mEos2 color channel; further confocal overview images from which the relevant fluorescence was identified are shown in *SI Appendix*, Fig. S11.

Neurotransmitter receptors and other proteins in the postsynaptic terminal of excitatory synapses are physically organized by scaffolding and structural proteins including PSD95 (30) and actin (31). We further evaluated the 2D MINFLUX performance for imaging actin and PSD95 in the mouse brain tissue at different imaging depths (Fig. 4).

In a subset of neuronal dendrites, actin was labeled by viral injection of LifeAct-EYFP, which was then recognized by indirect immunofluorescence postfixation with Alexa Fluor 647 as the fluorescent dye. LifeAct, labeling G- and F-actin, allows to visualize the morphology of the imaged neuron. In practice, we were able to acquire confocal previews in the EYFP and the Alexa Fluor 647 channel prior to MINFLUX measurements, which allowed to ascertain the structure of interest with reasonable confidence and exclude spots of apparent unspecific labeling. Judging from the confocal images acquired, there was indeed unspecific labeling visible in the Alexa Fluor 647 channel (but absent in the corresponding regions for EYFP).

MINFLUX, benefitting from the long-term stabilization with the focus-lock system (*SI Appendix*, Fig. S8), visualized small structures of characteristic morphology based on the actin content (Fig. 4*A*), that can be roughly divided into a spine head (as part of the synapse) and a spine neck connecting the synapse to the dendrite. As a clear border of the spines in the MINFLUX images could be identified, we delineated the spines and rejected outliers from unspecific antibody labeling or detached dyes for improved visualization of the data (*SI Appendix*, Fig. S9).

To approach the aim of measuring quantitative nanometer-precise protein distributions in living tissue, we thus performed MINFLUX on PSD95 endogenously labeled by mEos2 (32) in different imaging depths (Fig. 4*B*). With a FP directly fused to the PSD95 molecules, we no longer observed unspecific labeling. mEos2 is a photoconvertible fluorescent protein. Before photoconversion (in its green-emitting form), we excited it at 511 nm wavelength to find ROIs containing PSD95. Then the mEos2 proteins were photoconverted individually with the 405 nm laser light and subsequently excited at 560 nm in their orange-emitting form. We initially checked the photoconversion behavior in confocal imaging for each new tissue slice.

In the MINFLUX acquisitions of PSD95-mEos2 (parameters in *SI Appendix*, Tables S1 and S2), the emission events of the FP were confirmed to be brighter and more stable in the presence of an appropriate buffer [a 50 mM TRIS buffer in deuterium (33, 34), or the aforementioned blinking buffer] compared to the emission events in standard buffers such as PBS or ACSF. On average, the FP mEos2 delivered ~19% fewer fluorescence photons and a ~81% lower photon emission rate than Alexa Fluor 647 and exhibited different switching kinetics. Therefore, we used modified parameters for the postprocessing of the collected photon traces. The pinhole allowed to realize suitable SBR for single-fluorophore detection (*SI Appendix*, Fig. S10). A comparison of the fluorophore-adapted parameters is shown in *SI Appendix*, Table S2. Confocal overview images are presented in *SI Appendix*, Fig. S11.

Similar trends to the Caveolin-1 samples, at similar performance with depth, were observed for actin and the FP-labeled PSD95 regarding the fraction of valid localizations, SBR, and attained localization precision (Fig. 4 *C*–*E*). We note that, at ≲10 nm in up to 50 µm, the demonstrated localization precision was slightly worse for mEos2 than for Alexa Fluor 647. This can be mainly explained by localizing with half as many (~1,000) photons (mEos2) compared to the dye case, yielding ~$\sqrt{2}$ worse localization precision.

Compared to the earlier caveolin imaging, higher numbers of fluorophores in the ROI and therefore increasing MINFLUX measurement times for the sequential scanning posed additional high demands on the focus lock system that needs to be stable over the whole measurement time. Valid localization returns are reduced away from the glass due to increased background from a higher number of emitters out of focus. Therefore, the time expended was further prolonged (to tens of minutes in case of the PSD95 images and up to 1 to 2 h in the actin images).

Taken together, the MINFLUX data of caveolae and dendritic spines show that 2D MINFLUX with single-digit nanometer localization precision is possible in several tens of micrometers depth. As shown for PSD95-mEos2, this MINFLUX performance can also be obtained at the bluer wavelength and with a less bright emitter. A diversity of spine morphologies was resolved, with fine image detail resolvable (see the neck of the left spine in Fig. 4*A*). PSD95 localization patterns in the imaged examples were of a high diversity, reflecting both a putative structural heterogeneity and likely planar projection (see 3D imaging below).

### Live Tissue Imaging.

A FP expressed in the mouse provides the possibility to image in living tissue. As we expected severe movement due to breathing and heart beat in the living mouse (7), we performed MINFLUX imaging on acute tissue slices. We kept the slices in ACSF buffer and provided oxygen during measurements to keep the slices alive for several hours. Imaging in several depths in living tissue slices gave comparable results as in fixed tissue slices (examples of two depths in Fig. 4*F*). An only slightly worse SBR and localization precision at the same imaging depths compared to fixed-slice imaging can be rationalized by the less optimal buffer system for the FP in acute slices. Noticeably, the localization precision was slightly worse than in fixed slices even if the SBR was comparable or slightly more favorable. This slightly worse localization precision was probably due to movement in the living tissue, including PSD95 mobility (35, 36). The measurement time for a MINFLUX image of PSD95 with a field of view of 3.2 × 3.2 µm^2^ was on average ~20 min.

### MINFLUX Nanoscopy with Two Excitation Wavelengths.

For imaging postsynaptic proteins in context, we established a robust multicolor imaging scheme, employing spectral separation both in detection (16) and by excitation wavelength. The aforementioned fluorophores featured clearly separated absorption and emission spectra for this task and allowed sequential localization. An example of a two-color acquisition of actin and PSD95 is shown in Fig. 4*G*. The dendrite can be readily assigned and the corresponding PSD95 cluster selected.

Here, we localized first PSD95 and then actin. The PSD95-mEos2 acquisition was therefore not affected by the actin imaging. Since both fluorophores can be activated by 405 nm light, the second image may be affected by the first imaging round. This effect was not expected to be very detrimental. As the blinking dye can be switched on multiple times, we should register the majority of dyes in any case. Note that a more conservative approach would be to first image Alexa Fluor 647, without photoactivation, until no more events are obtained, then image mEos2, and then the dye again. We tested this approach for the 3D two-color imaging (see below).

### Analysis of 2D Distribution of VGlut1 and AMPA Receptors.

**Fig. 5.**
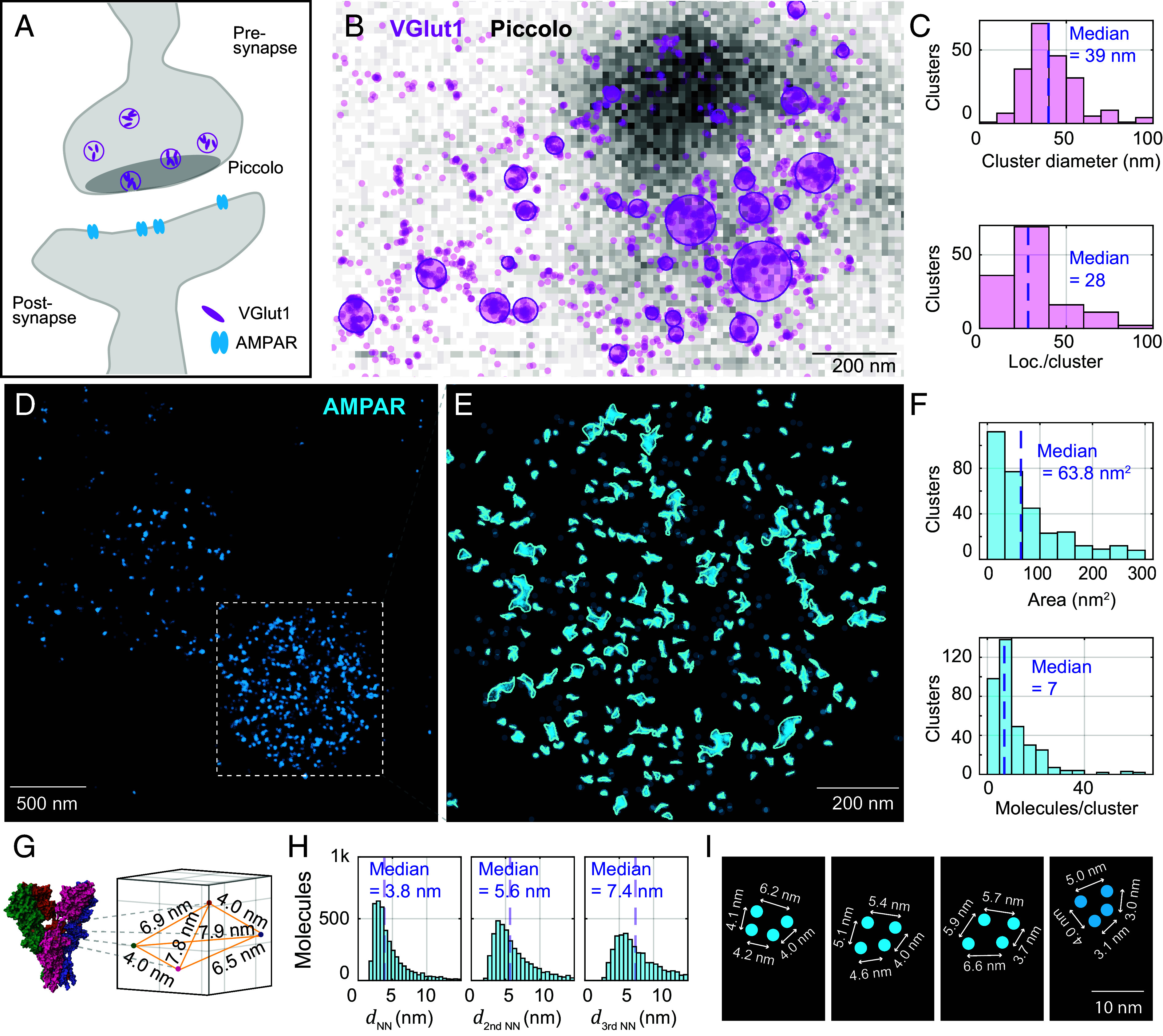
Cluster analysis of synaptic proteins in mouse brain tissue. (*A*) Schematic of a synapse showing the arrangement of VGlut1 in synaptic vesicles (presynapse), Piccolo situated close to the synaptic release site, and AMPA receptors (post-synapse). (*B*) Confocal image of Piccolo (grayscale) overlaid with MINFLUX acquisition of VGlut1 (localizations in transparent violet). VGlut1 is labeled by Alexa Fluor 647 with primary and secondary antibody. Clusters assigned by analysis with the *dbscan* algorithm are fitted with a circle, shown overlaid. (*C*) Cluster analysis of VGlut. (*D*) MINFLUX image reconstruction of AMPA receptors directly chemically labeled with CAM2-Alexa Fluor 647 conjugate. Localizations from the same emission event and localizations that fall within 2 nm of each other are assigned to the same molecule (AMPAR subunit). Molecules are plotted as cyan dots. (*E*) Zoom-in to the image region with highest molecule density, and cluster analysis of the AMPA receptors (clusters identified by *dbscan* and their border delineated using a spline-fit). (*F*) Cluster analysis of AMPAR. (*G*) AMPA receptor structure from PDB file 3KG2, and extracted distances between the labeled amino acids. (*H*) Distances *d_NN_* between AMPAR subunit localizations (nearest neighbors, second-nearest neighbors, and third-nearest neighbors). (*I*) Selected molecule geometries assumed to be part of completely labeled AMPA receptors.

Further molecular components of the synapse can be visualized. For illustration, we chose to image the arrangement of VGlut1 in synaptic vesicles of the presynapse (37) and AMPARs (38) in the membrane of the postsynapse (Fig. 5*A*). Piccolo, a protein situated close to the synaptic release site, served as context for a MINFLUX acquisition of VGlut1 (Fig. 5*B*). In these experiments, VGlut1 was labeled by Alexa Fluor 647 with primary and secondary antibody (confocal overview images in *SI Appendix*, Fig. S12). The apparent clustering was quantified further, with clusters assigned by analysis with the *dbscan* algorithm and shown by overlaid circles. Results of the VGlut1 cluster analysis (Fig. 5*C*) indicated a median diameter of 39 nm and contained ~28 localizations on average.

The AMPA receptor distribution was visualized by direct labeling with a chemical AMPAR modification (CAM2) probe conjugated to Alexa Fluor 647 (38). The probe labels three out of four possible AMPAR subunits, specifically GluA2, GluA3, and GluA4, but not GluA1. Functional AMPARs are tetramers, composed differently from GluA1-GluA4. Homogenous AMPARs containing only one subunit type and heterogenous AMPARs containing several subunit types are possible (39, 40). Up to a maximum of 4 sites (subunits) could therefore be labeled. From structural data of the AMPA subtype ionotropic glutamate receptor [PDB 3KG2, (41)], the spatial arrangement of the amino acid in the AMPAR targeted by labeling was extracted for reference (Fig. 5*G*).

In analyzing the MINFLUX recordings, localizations from the same emission event and localizations that fall within 2 nm of each other were assigned to the same AMPAR subunit (Fig. 5*D*). The image region with highest molecule density, putatively the post-synapse, was subjected to a cluster analysis by the *dbscan* algorithm, and cluster borders were delineated using a spline-fit (Fig. 5*E* and *SI Appendix*, Fig. S13). AMPAR clusters featured a median cluster area of ~60 nm^2^ and approximately 7 (median) fluorophores (i.e., labeled AMPAR subunits) present per cluster (Fig. 5*F*). Intriguingly, the distances between AMPAR subunit localizations—the average nearest neighbors, second-nearest neighbors, and third-nearest neighbors (histogram peaks at ~4 nm, ~5 nm, ~7 nm) quantified across the entirety of subunit positions in the field of view—were found to correspond approximately with the distances extracted from structural data (Fig. 5*H*). A comparison with simulations of AMPAR subunit localization yielded similar nearest neighbor distributions (*SI Appendix*, Fig. S14 and *Materials and Methods*). This shows that the ultraprecise nature of MINFLUX localization, even under the experimentally challenging tissue circumstances, rendered statistically valid mutual distances that were quite consistent with the expected intramolecular spacings. Individual patterns illustrate how, for a few instances of collections of sufficient points, fully labeled tetramers may be annotated (Fig. 5*I*). While STORM imaging (42) in conjunction with primary and secondary antibody labeling allowed to estimate spacings of tens of nanometers, the MINFLUX nanoscopy resolution is substantially better, allowing to interpret <10 nm distances. The close agreement between the distances expected from the structural and the MINFLUX data is only possible due to negligible linkage error by the direct chemical labeling. The structures that were recognized as nanodomains in earlier studies (43, 44) are dissected into smaller clusters composed of one or a few AMPA receptors (Fig. 5 *G*–*I* and *SI Appendix*, Fig. S15).

### 3D MINFLUX Scheme for Tissue Imaging.

With ~600 nm extent projected along the optical axis in the 2D MINFLUX imaging without depth discrimination, there was a clear potential for misclassification of PSD95 proteins that appear to localize on the dendrite but could also be positioned above or below. Quantification of distances among proteins in 2D is inherently prone to projection errors. We therefore chose to explore 3D localization for further two-color studies.

**Fig. 6.**
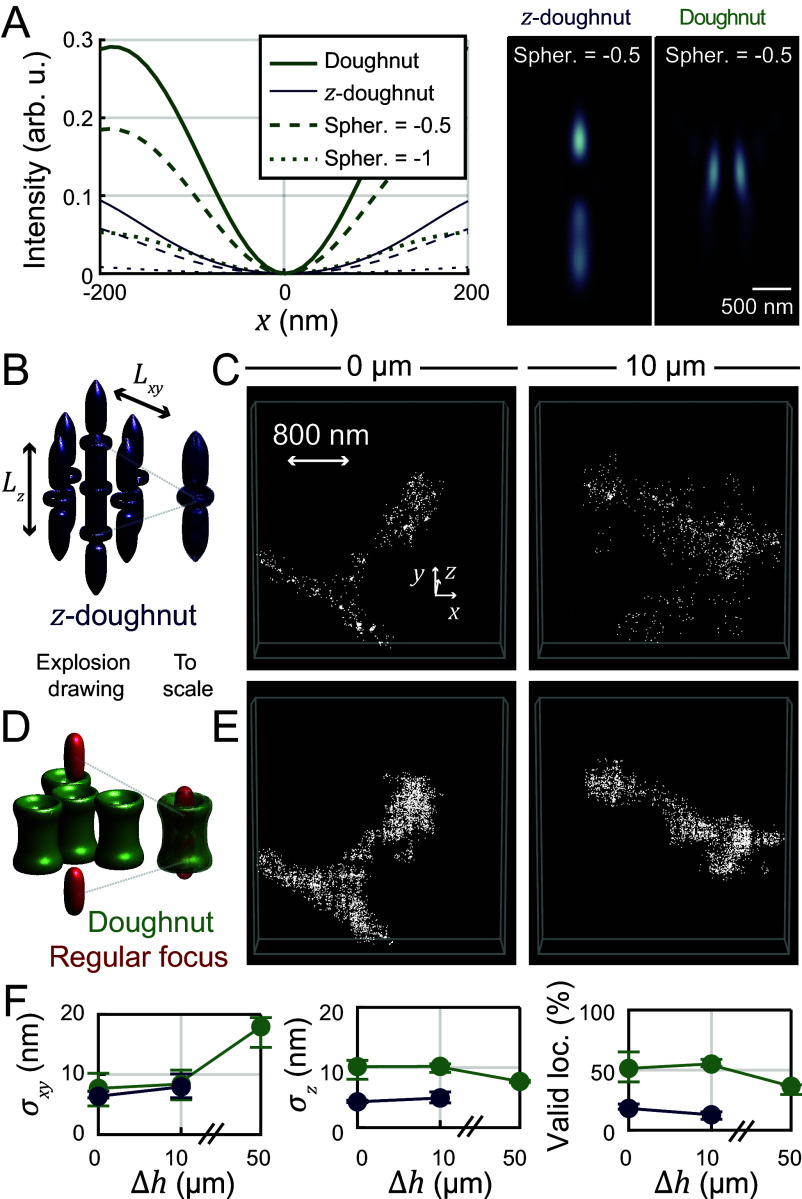
3D MINFLUX localization scheme for imaging in aberrant media. (*A*) Simulated focal intensity profiles for different spherical aberrations (Zernike coefficient of first-order spherical aberration; see *SI Appendix*, *Supplementary Text*). While the *z*-donut provides good imaging contrast along the optical axis, its contrast perpendicular to the optical axis is notably worse than for the donut, especially in the case of spherical aberrations. (*B*) Standard 3D MINFLUX localization scheme based on the *z*-donut. (*C*) 3D MINFLUX images of spines in 0 and 10 µm imaging depth in tissue acquired with the *z*-donut. (*D*) Donut/regularly focused beam 3D imaging scheme. (*E*) Same regions of interest as in (*B*) acquired with the donut/regular focus imaging scheme. (Interleaved acquisition: first *z*-donut, then donut/regular focus, then again *z*-donut). (*F*) MINFLUX metrics (color-coded) for 3D imaging with donut/regular focus or *z*-donut in different imaging depths.

In prior reports of the first 3D MINFLUX demonstrations (16, 17, 19), a “*z*-donut” which forms an intensity minimum in three dimensions was used for localization. The intensity gradient of such a *z*-donut is inevitably stronger along the optical axis than in the perpendicular direction. Further, the minimum (zero) definition contrast, especially perpendicular to the optical axis, decreases rapidly if there are sample-induced aberrations (9) (compare *SI Appendix*, Fig. S6). The zero definition is laterally considerably less robust (deep) in tissue than the zero of the classical donut (Fig. 6*A*). Transverse intensity profiles through simulated focal distributions illustrate this effect with increasing spherical aberration strength.

In the absence of tissue homogenization or clearing methods, we chose to implement and explore a 3D localization pattern consisting of two excitation beam shapes: the regular focus for localizing along the optical axis and the donut (“vortex”) beam for localizing perpendicular to it. This 3D localization pattern was utilized for the last iteration step of the iterative MINFLUX scheme. The previously described *z*-donut-based iterative MINFLUX scheme and our new donut/regular focus-based iterative MINFLUX scheme are illustrated in Fig. 6 *B* and *D*.

Choices of parameters of the modified least mean squares (mLMS) estimator for the *z*-donut localization were guided by previous work (16) and adapted for a bigger localization range along *z* (detailed in *SI Appendix*, Table S3). For the donut-regular focus scanning pattern, mLMS estimator parameters were found by repeatedly localizing the center of mass of fluorescent beads with the calibrated setup, displacing them by known distances with the fine stage and checking that the estimated center of mass of the fluorescent beads moved by the expected distance.

We compared the performance of our 3D MINFLUX scheme to the *z*-donut by interleaved acquisition, observing higher valid-event rates on the same structure in dendritic spine actin samples (Fig. 6*E* vs. Fig. 6*C*). The lateral precision was comparable, whereas axial precision was about half (~10 nm) of the precision achieved with the *z*-donut. Valid localization rates were improved several-fold, enabling robust imaging and deep-tissue imaging up to 50 µm imaging depth (Fig. 6*F*). The results experimentally confirm the reasoning that while the z-donut provides rather good imaging contrast along the optical axis, in the presence of aberrations, the contrast in the lateral (*xy*) dimensions becomes notably worse than for the lateral donut.

### Investigation of PSD95 and AMPA Receptor Distributions in 3D on the Post-Synapse.

**Fig. 7.**
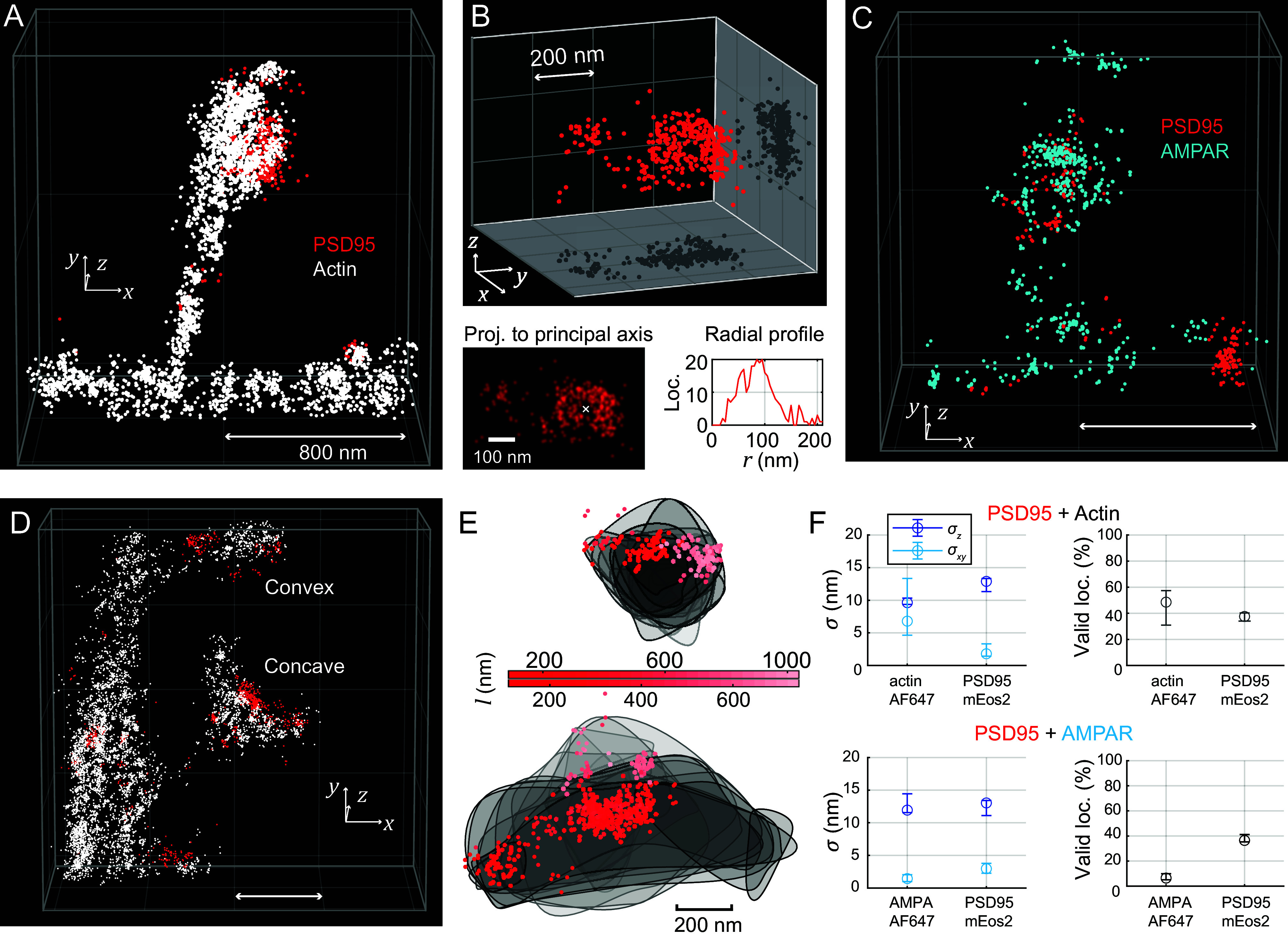
3D MINFLUX with two excitation colors for investigating the protein distributions on the post-synapse (spine head). (*A*) Region of interest showing part of a dendrite with a spine. Spine morphology is delineated similarly to the 2D two-color MINFLUX recording of PSD95/actin; localizations of actin in white, PSD95 in red. (*B*) More detailed view of the PSD95 distribution. Notably, PSD95 proteins are clustered and the biggest cluster is perforated. Projection of the localizations to an orthonormal head-on view, and radial profile centered on the hole in the cluster. (*C*) PSD95 and AMPA receptor (AMPAR) distribution on a spine with similar morphology. AMPA receptor localizations are plotted in blue. The highest AMPAR density is on the top of the convex spine head. (*D*) ROI containing two spines: one with a concave-shaped, one with a convex-shaped spine head. (*E*) Convex and concave spine head, shown projected from the top of the respective spine head. Gray lines delineate 80 nm thick slices of the actin dataset. PSD95 localizations shown as scatter, with the distance *l* along the line of sight from the top of the spine color-coded. (*F*) MINFLUX metrics over multiple 3D two-excitation-color MINFLUX images of actin and PSD95 as well as AMPAR and PSD95 (median values, whiskers represent interquartile range). Animations of the data in (*A*, *C*, and *D*) are found in Movies S1–S3. Scale bars: 800 nm or as indicated.

3D two-color MINFLUX (Fig. 7) enabled the mapping of protein localizations as 3D spatial distributions in context. We illustrate this with PSD95 along with actin on the postsynapse (Fig. 7 *A* and *D*), or with the distribution of AMPA receptors together with the distribution of PSD95 (Fig. 7*C*).

PSD95 arrangements may be analyzed in terms of their spatial extent and internal distribution. This is exemplified by a large group of PSD95 that is located in a rather large (~200 nm) diameter cluster which exhibits a central perforation (void) (Fig. 7*B*), as suitable projection of the 3D dataset and radial distribution analysis (radius ~90 nm) shows.

Spine heads of different morphologies, such as convex and concave shapes, were appreciable in imaged examples based on the volumes outlined by actin (Fig. 7*D*). PSD95 accumulations can be related in space to the underlying spine head geometry. For instance, a prominent PSD95 cluster sat distinctly on the side of a convex, bulb-shaped spine head (Fig. 7 *D* and *E* upper example), whereas the clustered PSD95 sat centrally on an elongated concave spine head (lower example in Fig. 7*D*, with oriented displays of data in Fig. 7*E*).

The complex distribution of the directly labeled membrane AMPARs was visualized as a further illustration of 3D capabilities in conjunction with PSD95. The AMPAR protein number density was appreciably highest at the top of the convex-shaped spine head (Fig. 7*C*). We applied an analysis based on the bivariate Ripley´s K function (45, 46) (*SI Appendix*, *Materials and Methods*) to investigate the relative arrangement of AMPAR subunits and PSD95 in our 3D two-color MINFLUX measurements in volumes that appeared to contain a postsynapse (*SI Appendix*, Fig. S16).

The data suggested a statistically relevant “anti-clustering” (i.e., spatial segregation) of AMPAR and PSD95 on all length scales below 400 nm (which is broadly the size scale of a postsynapse and spine head as a whole). The anticlustering, quantified as the dip in the variance-normalized function $H_{\mathrm{biv}}$ (*r*) (*SI Appendix*, Fig. S16*A*), indicating the most typical distances of separation, is especially pronounced for ~50 to 200 nm. Inspection of the 3D datasets (*SI Appendix*, Fig. S16 *C*–*K*) confirmed the in part stratified arrangements of PSD95 and AMPAR molecules. A model of a possible PSD95-AMPAR arrangement in the synapse, including the respective label-positions, is shown in *SI Appendix*, Fig. S16*B*.

While mutual distances of ~20 nm that appear statistically significant in the bivariate Ripley function can readily be rationalized for the “standard” connectivity of PSD95 and the AMPAR through an auxiliary transmembrane AMPAR regulatory protein (TARP) such as stargazin (47, 48), a substantial fraction of protein resides at larger separation. This would be the case for PSD95 molecules positioned farther away in the PSD, which has an extent in the orthonormal direction with the synaptic cleft of up to ~50 nm. PSD95 is known to link also to other postsynaptic receptors such as NMDA receptors, often clustered in the membrane adjacent to the center of the PSD, while AMPA receptors were observed more distributed over the extent of the PSD (49). In agreement with this previous finding, and as AMPARs and PSD95 are known to move in and out of synaptic sites 35, 50, many of the pairwise 3D distances between the two protein species over the volumes containing the PSD were found to amount to well above 50 nm.

With respect to quantitative performance, spatial precisions fell in the range of ~10 to 12 nm axially (Fig. 7*F*), and well below (~2 to 7 nm) in the lateral directions. The direct attachment of Alexa Fluor 647 to AMPAR led to shorter emission bursts and less robust localization success rates (lower valid localization fraction). At the same time, repeated on-blinks from individual copies of the dye likely contributed to a putatively rather complete sampling of the present dye molecules over the whole recording duration.

## Discussion and Conclusion

It goes without saying that any fluorescence microscopy, including MINFLUX, records nothing but the fluorophores. This obvious fact becomes highly relevant at the scale of the proteins themselves. Therefore, any conclusion drawn from the detection and localization of fluorophores has to take this fundamental limit of fluorescence imaging into account. Nonetheless, our initial 2D and 3D, two-color MINFLUX results in tissue, with <10 nm 3D fluorophore localization, open up broad avenues to investigate protein distributions on the single-synapse level in fixed and living brain slices. The ability of MINFLUX to differentiate by precise 3D localization both presynaptic and postsynaptic proteins involved in neurotransmission, learning, and memory within tissues at the nanometer scale opens up new possibilities for investigating the molecular mechanisms underlying behavior in natural and pathological states of the brain. The molecular heterogeneity of synapses (51, 52) within intact tissue contexts can potentially be assessed at few-nanometer resolution, particularly with anticipated advancements in (semi-)automated acquisition enabling the selection or automatic identification of ROIs based on specific experimental criteria.

The fact that single-digit nanometer localization precision is reached in the complex tissue environment is arguably remarkable. In this context, it is worth commenting that close-by fluorophores in the sample involve virtually identical optical paths through the sample and hence basically identical optical conditions, such as sample inhomogeneities. Any potential aberration-related spatial shifts in the minimum of the MINFLUX excitation pattern (which would lead to a shift in the spatial position of the recorded fluorophore) therefore affect close-by fluorophores identically. Thus, a faithful mapping of the relative position of adjacent fluorophores in the optically inhomogenous sample is ensured. This is why in MINFLUX nanoscopy the resulting nanoscale distributions of resolved molecular localizations can be interpreted with high confidence.

Our demonstrations further indicate that the use of a silicone oil immersion lens enables this MINFLUX resolution without dedicated online corrections to respond to sample-dependent depth aberrations. The absence of suitable bright reference objects (“guide stars”) at the required depth—which would be needed to perform an optimization of the excitation pattern at each new ROI—would make this more challenging to realize. Instead, the described results were obtained by setting up the optics based on calibration measurements at the coverslip surface, with no further adjustments at depth.

Nonetheless, looking ahead, the prospects for the development of online aberration correction highlight a further attractive feature of MINFLUX in this regard. In PALM/STORM, the localization relies completely on a large but inherently limited number of emitted fluorescence photons. This full reliance on emitted fluorescence makes it challenging to implement effective aberration correction methodology, as any correction approach inherently uses up emitted photons as well. In contrast, aberration correction in MINFLUX can be carried out by modifying the shape and direction of the stable and bright laser beam rather than the feeble fluorescence “beam” delivered by the fluorophore. The alternative but nonideal remedy of using an auxiliary laser beam for aberration correction would also not be needed.

As a point of particular interest, and as our PSD95 imaging demonstrates, MINFLUX imaging in tissue is also realized with fluorescent proteins, for which the number of emitted photons is more limited than for organic dyes but which are better compatible with living tissue. FP fusion proteins also inherently maintain a stoichiometric mapping of protein copy numbers in the microscopy analysis. An additional advantage of MINFLUX imaging in biological tissue is the need for comparatively low laser powers and only local illumination, which should be more compatible with imaging of living tissues over long periods of time.

## Materials and Methods

A custom-built MINFLUX nanoscope with a depth-adaptable focus lock system and progressive photoactivation capabilities utilized a silicone oil immersion objective lens for improved refractive index matching to the fixed or living tissue sample. Details of the optical setup, the sample preparation, fluorescence labeling strategies employed, and data acquisition as well as analysis can be found in *SI Appendix*.

## Supplementary Material

Appendix 01 (PDF)

Movie S1. **Animation of Fig. 7*A*.** The two-color 3D data of actin and PSD95 is rotated. Zoom-in to the post-synapse. Rotation of only PSD95 localizations.

Movie S2. **Animation of Fig. 7*C*.** The two-color 3D data of AMPAR and PSD95 is rotated. Zoom-in to the post-synapse and rotation of the post-synapse.

Movie S3. **Animation of Fig. 7*D*.** The two-color 3D data of actin and PSD95 is rotated. Zoom-in to two post-synapses and rotation of the PSD95 localizations.

## Data Availability

All study data are included in the article and/or supporting information.
