# Supplementary material for: MINFLUX fluorescence nanoscopy in biological tissue
Source: Proc Natl Acad Sci U S A. 2024 Dec 20;121(52):e2422020121. doi: 10.1073/pnas.2422020121 (PMC11670107; doi:10.1073/pnas.2422020121)
Supplement: Supplementary file 1 — Appendix 01 (PDF) [file pnas.2422020121.sapp.pdf]

PNAS

Supporting Information for
MINFLUX fluorescence nanoscopy in biological tissue

Thea Moosmayer[1,2*], Kamila A. Kiszka[1*], Volker Westphal[1], Jasmin K. Pape[1], Marcel Leutenegger[1], Heinz Steffens[1], Seth G. N. Grant[3], Steffen J. Sahl[1], Stefan W. Hell[1,4]

[1]Department of NanoBiophotonics, Max Planck Institute for Multidisciplinary Sciences, Göttingen 37077, Germany
[2]Georg-August University School of Science (GAUSS), University of Göttingen, Göttingen 37077, Germany
[3]Centre for Clinical Brain Sciences, The University of Edinburgh, Edinburgh EH16 4SB, United Kingdom
[4]Department of Optical Nanoscopy, Max Planck Institute for Medical Research, Heidelberg 69120, Germany

[*]T.M. and K.A.K. contributed equally to this work.

Corresponding author: Stefan W. Hell
Email: shell@mpinat.mpg.de

This PDF file includes:

Materials and Methods
Supporting Text
SI References
Figures S1 to S17
Tables S1 to S10
Legends for Movies S1 to S3

Other supporting materials for this manuscript include the following:

Movies S1 to S3

Materials and Methods

Animals

Animal procedures described here were carried out in accordance with institutional regulations on animal use in research. Experiments performed on living animals were approved and authorized by the Lower Saxony State Office for Consumer Protection and Food Safety (Niedersächsisches Landesamt für Verbraucherschutz und Lebensmittelsicherheit (LAVES)). Sacrificing rodents for subsequent preparation of living slices and cultures did not require specific authorization or notification (Tierschutzgesetz der Bundesrepublik Deutschland (TierSchG)).
All mice were housed with a 12 hours light/dark cycle and an *ad libitum* access to food and water.

Stereotaxic Injections

The protocol of stereotaxic injection has been described previously in (1, 2). Briefly, an adult mouse of C57BL/6J background was anesthetized by 1.0-2.0 % isoflurane (Isofluran CP, CP Pharma) in oxygen-enriched air (47.5 % oxygen, 50 % nitrogen, and 2.5 % carbon dioxide; Air Liquide) and fixed into a stereotaxic frame (SG-4N, Narishige). The scalp was incised and a craniotomy was performed on the parietal bone above the visual cortex of the left hemisphere. A pre-pulled, tapered, borosilicate glass injection capillary (World Precision Instruments, cat. 1B150F-4) was filled with a solution of pAAV-hSyn-Lifeact-EYFP virus (3) diluted 1:5 in sterile artificial cerebrospinal fluid (ACSF; NaCl 126 mM, KCl 2.5 mM, $CaCl_2$ 2.5 mM, $MgCl_2$ 1.3 mM, HEPES 27 mM, glucose 30 mM; pH 7.4). The capillary was subsequently lowered ca. 500 µm into the brain with an angle of 20° to the horizontal axis. A volume of 250–500 nl of virus-containing solution was injected using a pressure application system (TooheySpritzer, Toohey

Company) generating 30 ms pulses delivered with 20 psi on a manual command. The capillary was retracted and the scalp was surgically stitched by a polyamide surgical suture (6.0, Ethilon, cat. 697H). Subsequently, the animal was allowed to recover from anesthesia.
Perioperative analgesia was achieved by subcutaneous (s.c.) injection of Buprenorphine (Buprenovet, Bayer) and local analgesia of incision sites by s.c. injection of 2 % Lidocaine (Lidocainhydrochlorid 2 %, Bela-Pharm). Throughout the surgery, eyes were protected from dehydration by application of ointment (Bepanthen, Bayer) and a custom-built heating plate was used to maintain mouse body temperature. Analgesic and anti-inflammatory post-surgical care was achieved by s.c. administration of Carprofen (Rimadyl, Zoetis).

Intracardial perfusion fixation and fixed brain slice preparation

Intracardial perfusion took place following the period of around 4 weeks from the time of stereotaxic injection. Injected mice were anaesthetized by intraperitoneal injection of an overdose of Ketamine (Ketamin 10 %, Bela-Pharm) and subsequently transcardially perfused with Phosphate Buffered Saline (PBS, pH 7.4), followed by 4 % PFA in PBS (pH 7.4). The brain was dissected and post-fixed by an overnight incubation in 4 % PFA/PBS at 4°C. Afterwards, the fixative was removed, the brain was transferred into PBS (pH 7.4) and sliced into 60-120 μm thick consecutive coronal or sagittal sections using a vibratome (VT1200S, Leica).

Immunohistochemistry

Fixed slices were successively washed two times with Tris Buffer (TB), Tris Buffer Saline (TBS) and Tris Buffer Saline containing 0.5 % Triton-X 100 (TBST) (all with pH 7.6) for 15 min each at room temperature (RT). The slices were subsequently blocked with 10 % normal goat serum (NGS; Jackson ImmunoResearch, cat. 005-000-121) and 0.25 % bovine serum albumin (BSA) in TBST (blocking solution) for 1.5 h at RT. The tissues were then incubated with primary antibodies diluted in blocking solution for 72 h at 4°C on a rocking plate (primary antibodies used here: either anti-GFP (dilution 1:300; Abcam, cat. ab6556), anti-Caveolin-1 (dilution 1:100, Cell Signaling, cat. 3267) or anti-VGlut1 (dilution 1:200; Synaptic Systems, cat. 135 308). Additionally, slices labelled with anti-VGlut1 antibody were co-stained with anti-Piccolo antibody (dilution 1:300, Synaptic Systems, cat. 142 104). The slices were washed 4 times with TBST for 15 min each at RT and stained with Alexa Fluor 647-conjugated goat anti-rabbit secondary antibody (Thermo Fisher Scientific, cat. A-21245) diluted 1:1000-1:10 000 in TBST for 4 h at RT on a rocking plate. Slices co-stained with anti-Piccolo antibody were additionally labelled with Alexa 532-conjugated donkey anti-guinea pig secondary antibody (Dianova, cat. 706-005-148) diluted 1:500 in TBST. Slices were successively rinsed 2 times with TBST, TBS and TB, and stored at 4˚C in TB buffer until nanoscopy.

Injection of beads into fixed brain slices

A volume of 250-500 nl of PBS solution containing TetraSpeck fluorescent beads with average size of 100 nm (dilution 1:200; Thermo Fisher Scientific, cat. T7279) was injected into the 120 μm-thick, fixed coronal brain slice using the TooheySpritzer pressure application system. The bead solution was injected into several locations to cover the full depth of the slice.

Preparation of agarose-sucrose-bead sample

Agarose-sucrose pads were prepared similarly to the method described in (4). In short, a volume of 750 μl of distilled water was mixed with 8-24 mg agarose (low gelling temperature; Sigma-Aldrich, cat. A6560-25G) and 318-680 mg sucrose. The whole sample was weighed. Under constant stirring, the sample was warmed up to 100°C, held at this temperature for 2 min, then cooled down and held at 80°C for 2 min. Distilled water was added to fill up to the previous weight of the sample. Then, a volume of 250 μl of 100 nm size TetraSpeck fluorescent beads (Thermo Fisher Scientific, cat. T7279) diluted 1:200 in water was added to the sample while stirring on the heating plate. A cavity slide and a coverslip (thickness: 170 ± 5 μm) were heated up to 80°C. A volume of around 70 μl of prepared solution was pipetted into the cavity simultaneously sliding the coverslip over to avoid air bubbles in the sample. The rest of the solution was poured onto a slide, cooled down to RT, detached from the slide and used for refractive index measurement with the refractometer (Schmidt + Haensch, ATR-L).

Preparation of living brain slices

Living slices were prepared of brains isolated from adult PSD95-mEos2 C57BL/6J or CD-1 mice of both genders (5). Mice were sedated with isoflurane in a sealed container and quickly euthanized by cervical dislocation. Brains were dissected instantly, transferred into ice cold ACSF (pH 7.4) infused with 95 % O_2 and 5 % CO_2 and sliced into 300 µm thick coronal sections using a vibratome straight away. Prepared slices were either stained with the CAM2-Alexa Fluor 647 compound (6) or directly imaged with the MINFLUX nanoscope.

Chemical labeling of AMPA receptors

The protocol of chemical labeling of α-amino-3-hydroxy-5-methyl-4-isoxazolepropionic acid receptors (AMPAR) was adapted from (6). In brief, living brain slices were stained with 1 µM CAM2-Alexa Fluor 647 in ACSF (pH 7.4) infused with 95 % O_2 and 5 % CO_2 for 1 h at RT on a rocking plate. The slices were then washed 3 times with ACSF and fixed for 3 h with 4 % PFA in PBS (pH 7.4) immediately after washing. Fixed slices were rinsed 3 times with PBS (pH 7.4) and stored at 4°C until nanoscopy.

Synthesis of CAM2- Alexa Fluor 647 conjugate

The CAM2-Alexa Fluor 647 conjugate was synthesized following the method described in (6) and (7) with minor modifications.

For the synthesis of ethyl [7-amino-2,3-dioxo-6-(trifluoromethyl)-3,4-dihydroquinoxalin-1(2*H*)-yl]acetate**,** dimethylacetamide instead of dimethylformamide (DMF) was used as a solvent to improve purity of the reaction and increase yield of the product.

Briefly, 15 mg of 10 % Pd/C was suspended in 0.2 ml of dimethylacetamide in a heated 10 ml Schlenk flask filled with argon. Subsequently, hydrogen was introduced and the reaction was stirred for 30 min under a hydrogen atmosphere. A solution of 100 mg of ethyl [7-nitro-2,3-dioxo-6-(trifluoromethyl)-3,4-dihydroquinoxalin-1(2*H*)-yl]acetate in 1ml dimethylacetamide was then added from a flask filled with argon using a cannula (rinsed with 0.2 ml dimethylacetamide) and the reaction was stirred for 26 h at RT under a hydrogen atmosphere. After completion of the reaction, argon was passed through for 10 min, the reaction mixture was filtered over a small amount of Celite and washed 3x with 1 ml dimethylacetamide. The solvent was then removed on the rotary evaporator and 92.9 mg of crude product was obtained as a light-yellow solid. The crude product was used for the next reaction step without further purification.

Finally, the step of the deprotection of BOC-protected CAM was modified. To avoid hydrolysis, deprotection was performed with either HCl (Method A) or formic acid instead of DCM/TFA (Method B).

Method A:
In short, 7.0 mg of CAM2-BOC were dissolved in 150 µl acetonitrile in a flask purged with argon. The reaction mixture was subsequently cooled to 0°C in an ice bath and 15 µl of 4 M HCl in dioxane were added. The reaction was stirred for 2 h at RT. The progress of the reaction was monitored by HPLC. To complete the reaction, 50 µl of ACN were added after 2.5 h, 3 µl of 4 M HCl in dioxane were added at 0°C after further 2.5 h and additional 100 µl of ACN at 0°C and 100 µl of distilled water at 0°C after 3 h. After completion of the reaction, another 500 µl of distilled water were added at 0°C. The reaction mixture was then frozen and lyophilized to obtain 6.45 mg of CAM2-amine as a colorless solid. The resulting CAM2-amine compound was used for the next coupling steps to the Alexa Fluor 647-NHS-ester without further purification.

Method B:
Briefly, 4.0 mg of CAM2-BOC were stirred in 250 ml formic acid for 45 min. After completion of the reaction 20.3 µl of distilled water were added at 0°C, the reaction mixture was frozen and lyophilized. To remove excess of formic acid 203 µl of distilled water were added, the reaction mixture was frozen and lyophilized. This procedure was repeated twice to receive 3.7 mg CAM2-amine as a colorless solid, which was used in the next coupling steps without further purification.

Cells

U-2 OS cells (ECACC, cat. 92022711, lot 17E015) were cultured in modified McCoy's 5A medium (Thermo Fisher Scientific, cat. 16600082) supplemented with 10 % (v/v) FBS (Bio&Sell, cat. S0615), 1 % (v/v) Sodium Pyruvate (Sigma, cat. S8636) and 1 % (v/v) Penicillin-Streptomycin (Sigma, cat. P0781) in a humidified 5 % CO_2 incubator at 37°C. Cells were seeded on coverslips 24 h before staining.

Immunocytochemistry

U-2 OS cells were washed with PBS and subsequently fixed with 8 % (w/v) PFA in PBS for 5 min at 37°C. Cells were then permeabilized with 0.5 % (v/v) Triton-X in PBS for 5 min at RT and blocked with 2 % (w/v) BSA in PBS (blocking solution) for 10 min at RT. Afterwards, cells were incubated with primary rabbit anti-Caveolin-1 antibody (Cell Signaling, cat. 3267) diluted 1:200 in blocking solution for 1 h at RT. Subsequently, cells were rinsed with blocking solution and incubated with Alexa Fluor 647-conjugated goat anti-rabbit secondary antibody (Thermo Fisher Scientific, cat. A-21245) diluted 1:5000 and 1:7000 in blocking solution for 1 h at RT. Cells were then washed with PBS (pH 7.4) and stored at 4°C until nanoscopy.

MINFLUX nanoscope for tissue samples

A MINFLUX nanoscope for imaging in tissue was built based on the nanoscope design described in (8, 9). A detailed overview of the confocal beam-scanning MINFLUX setup is given in Fig. S1.

The nanoscope is equipped with a 100x silicone oil immersion objective (NA = 1.35; UPLSAPO100XS, Olympus) and a fully computer-controlled sample positioning system. The sample positioning system consists of a coarse (50 nm step size) stepper-motor z-stage (M-230.25, Physik Instrumente (PI)) with an inductive LVDT (linear variable differential transformer) sensor for measuring displacement along the optical axis, a coarse (100 nm positioning resolution) xy-stage (M-686.D64, PI) and a fine (0.1 nm positioning resolution) piezo-driven xyz-stage (P-733.3DD, PI). An imaging-depth adaptable lock system (described in detail in the main text, Fig. 2) for stabilizing the sample position on the nanoscope was built in close proximity to the objective lens.

Excitation and activation lasers with their ventilation and their power modulation are mechanically decoupled from the main setup (see Fig. S1*D*). The setup contains three cw excitation lasers at 647 nm, 560 nm and 511 nm respectively (2RU-VFL-P-2000-647-B1R and 2RU-VFL-P-5000-560-B1R, MPB Communications; IBEAM-SMART-511-S-HP, Toptica) and the activation laser at 405 nm (Phoxx 405-60, Omicron) with corresponding power modulation and spectral selection possibility via acousto-optic modulators (AOMs, (MT110-A1-VIS)) and acousto-optic tunable filters (AOTFs, (AOTFnc-VIS-TN, AA Opto-Electronic)). The 511 nm laser has an integrated power modulation and therefore does not require an AOM. The three excitation laser beams are rearranged and coupled into polarization maintaining (PM) fibers, guiding the laser light to different out-couplers into the main setup (Fig. S1*A*). All three excitation wavelengths are coupled into the PM fiber for widefield illumination and a separate PM fiber for regular focus illumination. Activation light can be either added to the widefield PM fiber or guided separately via the activation beam PM fiber, allowing for a regular focus activation beam in the sample plane. For the (z-) doughnut illumination, the red beam (647 nm) and the green beams (511 nm and 560 nm) are coupled into separate PM fibers as the different colors require separate phase masks for beam shaping on the spatial light modulator (SLM, (SLM-100, Santec)). Here, chromatic focus shifts can be compensated, enabling precise two-color MINFLUX nanoscopy with up to three different excitation colors. Further, setup- and/or sample-related aberrations can be corrected by the SLM.

From the SLM, the laser light passes the beam scanning system and enters the objective lens. The beam scanning system consists of electro-optical deflectors (EODs, (M-311A AD*P, Conoptics)) for fast beam scanning over a small area perpendicular to the optical axis (xy), the tip/tilt piezo mirror (TM, (PSH 10/2 SG, Piezosystem Jena)) for (de-)scanning a bigger field of view (in xy) and the deformable membrane mirror (DMM, (Multi-3.5, Boston Micromachines Corporation)) for fast z-(de-)scanning. Several $4f$-relay lens systems adapt the beam diameter and relay the scanning system pupils into the back aperture of the objective lens. Activation light is coupled in after the EODs. A dichroic mirror (DM, (zt440/514/561/640rpc, Chroma)) reflects the activation and excitation wavelengths towards the objective and transmits the fluorescence emission to the detection. A motorized DM (ZET405/514/647 TIRF, AHF) placed between DMM and objective allows the switching from the confocal or MINFLUX imaging mode to the widefield imaging. Motorized achromatic quarter- and half-wave- plates (mQWP, mHWP, (RAC 3.2.15, RAC 4.4.15, B. Halle)) were used to control the polarization of the excitation light in the back aperture of the objective.

The first detection box, directly behind the main DM, contains notch filters for blocking light from the excitation and activation lasers (ZET647NF, ZET561NF, ZET405NF Chroma and ZET514TopNotch, AHF) and a short-pass filter (ET750sp-2p, Chroma) for blocking light from the focus lock system. Motorized flip-mirrors are used to direct the fluorescence either onto the widefield camera, the large area detection avalanche photodiodes (LA APD) for point spread function (PSF) measurements or the confocal pinhole with variable size for spatial filtering and then onto the multimode fiber leading towards the multicolor detection. The LA APD has a detection area of >> 1 AU (non-confocal APD).
The fluorescence light that passes through the first detection box (Fig. S1*C*), is spatially filtered by the variable confocal pinhole (MPH16, Thorlabs), coupled into a multimode fiber (M50L02S-A, Thorlabs) and coupled-out in the Multicolor-Detection (Fig. S1*B*). In the Multicolor-Detection, fluorescence light is spectrally separated into a green, an orange and a red spectral channel by DMs (FF560-FDi01, FF640-FDi01, Semrock). Fluorescence light in the three resulting spectral channels, is split by customized tunable filters (TSP-01-561-25x36, TSP-01-625-25x36, TSP01-704-25x36, Semrock) that can be spectrally tuned by computer-controlled servo motors to a custom-chosen ratio, and imaged onto the detection avalanche photodiodes (APDs, (SPCM-AQRH-13-TR, Excelitas Technologies)). For good isolation from ambient light, two layers of light-proofing boxes are built around the Multicolor-Detection box. The rest of the setup is housed in with one light-proofing layer.

Nanoscope control software

We adapted the custom written LabView programs from previous MINFLUX implementations (8, 9). The focus lock software was augmented to include the control of the adaptive lens and the absolute-positionable mirror (AG-M100D, Newport). A control system for the servo motors moving the half-wave plate and the quarter-wave plate was added to the main MINFLUX user interface. This allows to optimize the circular polarization for different excitation colors. A new 3D localization pattern was implemented, combining regular focus and doughnut exposures. A functionality to ramp up the activation intensity was added (described in the main text and Fig. 2).
A control unit for the DMM for focus-scanning consisting of a field-programmable gate array (FPGA, (PCIe-7852R, National Instruments)) and a Camera-Link interface was linked to the main FPGA (USB-756R, National Instruments). The host program for user interface of the DMM FPGA and the code for the DMM FPGA were written in LabView (LabView 2020 sp1, 32bit).

MINFLUX data acquisition

Data were acquired following the steps described in (9) with some adaptations. The widefield fluorescence image was used to choose the general region of interest (ROI). Due to the better optical sectioning in confocal mode, the ROI for MINFLUX acquisition was selected based on confocal overview scans. Because Alexa Fluor 647 is in an emitting state at the outset, the structure of interest can be seen by acquiring a confocal or widefield image before the MINFLUX measurement. In case of the fluorescent protein mEos2 the ROI was selected by imaging the green-emitting form. Before MINFLUX acquisition, Alexa Fluor 647 was switched-off by applying excitation light at 647 nm. Activation and probing of the fluorophore emission were started by scanning with steps of 150-200 nm to homogenously activate or photo-convert fluorophores within the ROI. Emitting molecules were iteratively centered and localized with the modified least mean squares (mLMS) estimator (as described in (8)) implemented on the FPGA. The variable pinhole was used to adapt the confocal pinhole size to optimize the MINFLUX metrics in the sample. For the statistics shown in Fig.4*C-E*, data are pulled together from the pinhole range 0.4-0.6 Airy Units (AU).
Sample-dependent measurement parameters for 2D MINFLUX are detailed in Table S2. For 3D MINFLUX acquisitions, parameters are shown in Tables S4 and S5. In the z-doughnut vs. doughnut/regular focus acquisition scheme comparison, the MINFLUX image employing the z-doughnut was acquired first, then the doughnut/regular focus scheme was run over the same field of view, then again, the z-doughnut scheme was used. The data sets with z-doughnut were combined.

Progressive activation

The imaging time per molecule can be divided into three parts:

1. t_{act}, the duration to activate the molecule,
2. t_{readout}, the duration to read-out the photons from the activated molecule,
3. t_{jump}, the time to move to the next location.

Step 1 and 2 are repeated until either no further activations are detected for a maximal duration $t_{\mathrm{act,\,max}}$ or a maximum imaging time $t_{\mathrm{image,\,max}}$ has been reached at the current location.

Typical values are: $t_{\mathrm{act,\,max}}$ = 2 s, t_{readout} = 0.1 – 1 s, t_{jump} = 200 ms.

t_{readout} cannot be further reduced due to the maximal photon flux that can be extracted from the molecule. Meanwhile, t_{jump} is due to the response time of the tip-tilt piezo mirror in the system. The probability to activate depends linearly on the dose of activation light applied and the density of molecules that can be activated within the activation volume. The activation intensity has to be low enough that even in regions of high densities of activatable molecules, the probability that more than one molecule is activated at the same time is low. This leads to a typical activation scheme where the activation intensity starts out low and is increased manually when the activation rate appears to be too low after the whole field of view has been imaged repeatedly and the density of activatable molecules had been reduced significantly.

This approach has drawbacks:

1. in regions with a low molecule density, t_{act} is very long and can often reach $t_{\mathrm{image,\,max}}$;
2. the user has to intervene constantly by increasing the activation power to keep the average activation rate reasonably high.

Therefore, we implemented an activation scheme that we are calling Progressive Activation. It starts with a brief period (e.g. 20 ms) of almost no activation (limited by contrast of the digital laser power modulation), to avoid additional activations in case a molecule is already activated. Then it continues at a low activation intensity (about 2 % of the final intensity) that ramps up exponentially to the maximum intensity (see Fig. S4*A*). The reason for the exponential increase is that for each time interval the activation probability is increased by the same factor. The time needed for constant (or homogeneous) activation to reach the same activation dose as progressive activation is estimated. Within 0.3 s the progressive activation would have applied the same activation dose as the homogeneous activation within more than 2 s (see Fig. S4*B*,*C*). This means in turn that the $t_{\mathrm{act,\,max}}$ of 2 s of activation previously needed at a given spot can be compressed into 0.3 s, and, if fluorophores to be activated are present, to an even shorter period of time.

Sample mounting

To correct for a drift of the sample during the MINFLUX measurement, all coverslips used for mounting were pre-treated with gold nanorods (Nanopartz, cat. A12-25-850-CTAB-DIH-1-25) employed here as fiducial markers. The nanorods were applied as described in (9). In brief, nanorods were diluted 1:3 in PBS, sonicated for 10-15 min at RT and incubated onto a high precision coverslip (170 ± 5 µm thick) for 10 min at RT. Afterwards the coverslip was rinsed several times with PBS to remove unbound nanorods.

Anaerobic redox blinking buffer containing 0.8 mg/ml glucose oxidase (Sigma-Aldrich, cat. G2133), 128 µg/ml catalase (Sigma-Aldrich, cat. C100-50MG), 50 mM Tris-HCl (pH 8), 10 mM NaCl, 10 % (w/v) glucose and 30-50 mM MEA (cysteamine hydrochloride; Sigma-Aldrich, cat. M6500) was used for MINFLUX imaging of fixed samples stained with Alexa Fluor 647, following the procedure described in (9). Two-color images of Alexa Fluor 647 and mEos2 were also acquired using this buffer.

Fixed brain slices with PSD95 endogenously fused with the photoconvertible fluorescent protein mEos2 were imaged in 50 mM Tris buffer (pH 8) in 95 % D_2O to reduce the short time blinking and increase the photon count of mEos2 (10).

All fixed samples were sealed with dental glue (eco-sil speed, Picodent, cat. 1300 7100).

Living brain slices were imaged in a custom-built imaging chamber shown in Fig. S2. Briefly, 300 µm thick living slices were placed onto a high precision coverslip pre-coated with nanorods and pre-fixed to the chamber with eco-sil speed dental glue. Slices were secured by a plastic grid magnetically attached to the imaging chamber and imaged in ACSF infused with 95 % O_2 and 5 % CO_2.

MINFLUX data analysis of depth imaging series

Trace segmentation and position estimation were performed as described in (9), with the adaptation that the starting threshold for estimating signal emission was set automatically in the depth imaging measurement series. For automatic estimation of the starting threshold for signal emission, a quantile filter was set on the emission trace in the last iteration, which removes extreme outliers. Then, the remaining emission trace was fitted to a mixture of two Gaussians and the threshold between them was taken as optimal threshold between signal and background.

Emission events were classified, employing a Hidden-Markov-Model (HMM) to estimate the time-dependent state of the fluorophore ("off", "on" or "blinking") in the detection region from the photon trace. Emission that was considered valid, meaning that it was assigned the states "on" or "blinking", was segmented into groups of 1000 or 2000 photons (thus guaranteeing unbiased position estimation) for separate localizations, from which experimental estimates of the localization precisions were also obtained. The localization precision was determined as the standard deviation of a Gaussian fit to the position deviation of the localizations from the mean position of the molecule localized in x and y.

Classified events affected strongly by out-of-focus fluorescence could be identified by high $p_0$values close to 0.25, which is equivalent to no intensity modulation from moving the excitation doughnut through the TCP. Clear separation between centered molecules and background was possible by monitoring the time-averaged p_0 value. A sharp increase in the p_0 value to an average of 0.25 coincided with off-switching steps. This was the case for most molecule events and the p_0 value was not required as an identifier for valid states here, because the HMM already classifies the event correctly. However, especially in structures with a high label density, events that were strongly affected by out-of-focus fluorescence were recorded. To discard such events (which the HMM might wrongly classify as valid states), the p_0 value was used as a quality criterion and all re-segmented photon bunches with $p_0 \geq 0.23$ were considered as background. This procedure inherently avoided artifactual localizations distributed along the scanned grid positions. Detailed post-processing parameters are listed in Table S6.

Cluster-analysis of the VGlut1 and AMPAR datasets

For the VGlut1 and AMPAR cluster analysis, we followed the post-processing procedure described in (9). Detailed post-processing parameters are shown in Table S7. In case of VGlut1, the cluster analysis was performed over localizations based on the re-segmented photon trace. In case of AMPARs clustering, molecules were assigned by combining re-segmented localizations if they came from the same emission event (following an activation) or if they were closer than 2 nm. The MATLAB *dbscan* algorithm (11), based on (12), was employed for assignment of clusters. For VGlut1, the parameters of the *dbscan* algorithm were set to epsilon = 20, MinPts = 15; for AMPARs to epsilon = 20, MinPts = 3. For VGlut1, a transparent circle was drawn over the localized clusters using the MATLAB function *viscircles* (13). For AMPARs the border of the clusters was interpolated using cubic splines (MATLAB function *cscvn* (14), based on (15)).

MINFLUX image rendering

2D MINFLUX images are rendered by plotting a Gaussian distribution for each localization (9). Overlapping Gaussian distributions are summed-up (non-linear colormap).

3D MINFLUX images are displayed as 3D scatter plots with a marker size of 15 nm (~3σ of the localization precision) or as isosurface renderings. For the isosurface rendering, the localization data was converted to a 3D histogram using the MATLAB function *histcn* (16) and the histogram data was rendered using the MATLAB *isosurface* function (17).

MINFLUX images of actin in dendrites are delineated following a two-step procedure. The first step is a nearest-neighbor filtering to reject single and isolated localizations with a mean distance of more than 150 nm to the next 20 neighbors. The nearest neighbor distances were calculated using the MATLAB *knnsearch* function (18), based on (19) with the number of nearest neighbors K = 20. The mean over the 20 nearest neighbor distances $\langle D_{NN} \rangle$ was calculated for each localization. Only localizations with $\langle D_{NN} \rangle < 150\,\mathrm{nm}$ were selected. In the second step, the structure was reconstructed using the MATLAB shape-reconstruction function 'alphashape' (20) on the filtered localizations with a custom-chosen alpha-radius α, that scaled anti-proportionally with the localization density in an image.

The shape reconstruction approach is illustrated in Fig. S9*B*.

Simulation of AMPA receptors

To simulate the 2D MINFLUX images of AMPARs, the positions of the CAM2 labeling site (LYS471) were extracted from the protein data base file 3KG2 (21). Randomly distributed AMPARs were simulated in a field of view of 1500 nm × 1500 nm × 100 nm with a minimum distance of 10 nm to each other. Between one and four subunits were labelled and free rotation of the receptor about the optical axis as well as up to ±45° rotation perpendicular to the optical axis was allowed. A Gaussian distribution of localizations around the receptor subunits with a sigma of 2 nm was simulated. Nearest neighbor histograms are plotted. The results are compared to a random distribution of dots without the AMPAR geometry and to the measured data (see Fig. S14).

Bivariate Ripley´s K function

We applied the bivariate, variance-normalized Ripley´s K function (22) for analyzing the relative arrangement of AMPAR subunits and PSD95 in our 3D two-color MINFLUX measurements. The bivariate Ripley´s K function is defined as

$$K_{i,j}(r) = \sum_{k} \sum_{l} \frac{I\left(d_{i,k}, d_{j,l} < r \right)}{k(i,j)\, \lambda_i \lambda_j\, V} \quad (1)$$

with the characteristic function I = 1 in case its argument is true and I = 0 in case its argument is false. $k(i,j)$ is an edge correction factor, λ_i ist the density of points of type i and V is the volume taken into consideration.
The Ripley´s K function (equation (1)) can be variance-normalized

$$H_{biv}(r) = \sqrt{\frac{K_{i,j}(r)}{\pi}} - r. \quad (2)$$

We manually selected volumes for analysis that appeared to contain at least one post-synapse (examples in Fig. S16*C*-*K*). We calculated the 99 % confidence interval (CE) by Monte Carlo simulations as in (23), and correspondingly normalized it to the interval [0,1]. The results obtained are shown in Fig. *S*16*A*.

Supporting Text

Focal intensity distribution in tissue

MINFLUX nanoscopy works by centering an excitation-beam minimum onto an emitter. Therefore, the shape of the excitation beam (its intensity distribution) needs to be known. When imaging deep into tissue, the excitation light as well as the fluorescence interact with the tissue matter, which can influence the amplitude, phase and polarization. In MINFLUX nanoscopy, the fluorescence photon rate is measured, which is proportional to the excitation intensity. We can estimate the position of the molecule from the number of photons detected when the excitation beam is at each position in the targeted-coordinate pattern (the scan-pattern).

Modeling of depth-induced aberrations by Zernike polynomials

Assuming that the tissue slice is a homogeneous optical layer with a certain isotropic refractive index and a flat surface, it only influences the phase of the photons. In this simplified model, we can calculate the PSF shape in different depths in tissue.
We compare the situation when imaging directly on the coverslip to the situation when imaging in tissue by moving the sample towards the objective by a certain distance Δh.
In the optical image, the light rays that come from the objective travel through the immersion medium with refractive index n_1 and hit the coverslip with an angle α that is dependent on the radial position ρ of the rays in the back aperture. From Snell's law, we can calculate the angle γ in the tissue sample with refractive index n_3:

$$\gamma = \text{asin}(n_1/n_3 \cdot \sin(\alpha)). \tag{3}$$

The angle α is given by radial ray starting position ρ in the objective back aperture and the focal length f of the objective

$$\alpha = \tan^{-1}\left(\frac{\rho}{f}\right). \tag{4}$$

When moving the objective lens by the distance Δh through the immersion oil towards the sample or when increasing the thickness of the coverslip by Δcs, the ray crossing point shifts by the distance $\Delta h_{\text{optical}}$ into the sample (compare Fig. S17):

$$\Delta h_{\text{optical}} = \frac{\tan(\alpha)}{\tan(\gamma)} \cdot \Delta h + \frac{\tan(\beta)}{\tan(\gamma)} \cdot \Delta cs. \tag{5}$$

Since we use coverslips with constant thickness, we set $\Delta cs = 0$.
In the case of central rays or no refractive index mismatch between sample and immersion oil, $\alpha \approx \gamma$ and the optical depth is equal to the mechanical depth. However, this is not the case for the outer rays if $n_1 \neq n_3$, which leads to broadening of the focus, because rays with different angles α constructively interfere at different depths in the sample. Therefore, the effect of focusing a distance Δh into the sample is equivalent to a phase shift $\Delta\varphi$ in the objective back aperture:

$$\Delta\varphi(\rho, \Delta h) = \frac{2\pi}{\lambda}\left(n_1\sqrt{\rho^2 + \Delta h^2} - n_3\sqrt{\rho^2 + \Delta h_{\text{optical}}^2(\rho, \Delta h)}\right). \tag{6}$$

With equations (3), (4) and (5), equation (6) can be simplified to:

$$\Delta\varphi(\rho, \Delta h) = \frac{2\pi}{\lambda}\Delta h\left(n_1 - \frac{{n_3}^2}{n_1}\right)\sqrt{1 + \frac{\rho^2}{f^2}} \; . \tag{7}$$

To get a more intuitive understanding of the influence of this phase shift onto the PSF of the microscope, we want to expand equation (7) into Zernike polynomials $Z_n^m(r,\theta)$ in cylindrical coordinates (24). Zernike polynomials are an orthogonal and complete set of functions defined on the unit circle and are commonly interpreted as optical aberrations.
Since the function $\Delta\varphi$ is radially symmetric, only the Zernike polynomials with angular index $m = 0$ need to be considered. These are the Zernike polynomials for piston, defocus, primary and higher-order spherical aberration.
We define $\rho = r\,\rho_{max}$, with $0 \leq r \leq 1$, and rewrite equation (7) to:

$$\Delta\varphi(r, \Delta h) = \frac{2\pi}{\lambda}\Delta h\left(n_1 - \frac{{n_3}^2}{n_1}\right)\sqrt{1 + r^2\frac{\rho_{max}^2}{f^2}}. \tag{8}$$

As the Zernike polynomials are orthogonal and complete, we can then calculate the Zernike coefficients $c_n^{0\prime}$ for $n = 0,2,4,6,8, \ldots$ via:

$$c_n^{0\prime} = 2\int_0^1 \Delta\varphi(r, \Delta h)\, Z_n^0(r)\, r\, dr. \tag{9}$$

The Zernike expansion can be done analytically, but it leads to rather lengthy terms.
Therefore, we just show the results for the numerical values $f = 1.8\ \text{mm}$ and $\rho_{max} = 2.43\ \text{mm}$:

$$\Delta\varphi(\mathrm{r},\,\Delta h)\;\approx\frac{2\pi}{\lambda}\Delta h\left(n_1-\frac{{n_3}^2}{n_1}\right)$$
$$\left(1.3688\,Z_0^0(r)+0.1938Z_2^0(r)-0.0125\,Z_4^0(r)+0.0016\,Z_6^0(r)\;-0.0003\,Z_8^0(r)\right).\qquad(10)$$

For comparison and validation of this approach, the "ground truth" phase function (equation (8)) is compared to its Zernike expansion up to order n = 8 (equation (10)).
A reconstruction using only piston, defocus and primary spherical aberration (n ≤ 4) gives agreement between the phase function and its Zernike expansion.
While piston, being only a constant phase offset, does not affect the PSF and the defocus only shifts the position of the PSF along the optical axis, the spherical aberration affects the shape of the PSF. The higher-order spherical aberrations contribute much less to the phase function than the primary spherical aberration and we therefore neglect them in the following. We measure the excitation PSF by scanning the excitation beam over a point-like object (i.e. a fluorescent bead or a small Caveolin-1-cluster). We can further determine the Zernike coefficient for spherical aberration by adjusting the Zernike coefficient for the phase correction of the excitation beam via the SLM until the PSFs no longer show the aberration linked to the respective Zernike polynomial. From equation (10), we obtain for the spherical-aberration Zernike coefficient in the objective back aperture:

$$c_4^{0\prime}\;=\;-0.0125\;\frac{2\pi}{\lambda}\;\Delta h\left(n_1-\frac{n_3^2}{n_1}\right).\qquad(11)$$

To compare with the experimental data, we normalize the Zernike coefficient $c_4^{0\prime}$ to k = $\frac{2\pi}{\lambda}$ and correct with a pupil magnification factor $m_p\,=\,(\frac{4}{3})^4$ since the SLM is imaged with a 4*f*-system with a focal length ratio of 3:4 onto the objective back aperture (the scaling factor is determined as described in (25)). We further multiply with a phase scaling factor $m_r\,=\,2$, since the SLM is used in reflection mode and therefore the phase correction is applied twice. Therefore, equation (11) is rewritten as:

$$\mathrm{c}_4^0=-0.025\;\left(\frac{4}{3}\right)^4\;\frac{2\pi}{\lambda}\Delta h\left(n_1-\frac{{n_3}^2}{n_1}\right),\qquad(12)$$

resulting in an equation that links the spherical aberration to the refractive index of the sample.

Calibrating refractive index measurements by spherical aberration determination

First, we measured the spherical aberrations in agarose-sucrose pads with embedded fluorescent beads. We determined the Zernike coefficient c_4^0 in different depths in the sample. The agarose-sucrose mixture allows us to tune the refractive index with the content of sucrose, similar to how it is described in (4). We measured the refractive index of the resulting sample using a critical–angle dispersion refractometer (Schmidt + Haensch, ATR-L; accuracy: 0.0005 RI at 20°C). Since the agarose-sucrose pad is homogeneous as well as transparent in the optical wavelength range and does not affect the polarization of the light, it is a good model system for testing the influence of the refractive index mismatch on the focal light intensity distribution. We used the silicone oil immersion objective for the PSF measurements. Example images of measured PSFs in 0, 40 and 80 µm imaging depths are shown in Fig. S6*G-I*. We determined the Zernike coefficient c_4^0 experimentally by adding a Zernike-based phase correction to the SLM until the aberrations visible in the PSF measurements with different phase masks are minimal. We fit a polynomial of first order to the spherical aberration coefficient over the depth. This is shown in Fig. S6*A-C*. From the gradient of the linear fit $m\,=\,\frac{c_4^0}{\Delta h}$, we can then calculate the refractive index of the sample by solving equation (12) for n_3:

$$n_3\;\approx -0.177\;\sqrt{-405\,m+32\,n_1^2}.\qquad(13)$$

The refractive index of the silicon oil immersion is $n_1\,=\,1.406$ at RT (as specified by the manufacturer). The refractive indices calculated from the PSF measurements with equation (13) for the samples shown in Fig. S6*A-C* are shown in Table S9. They match with the refractive indices measured by the refractometer.

This can be seen as a validation of our theoretical model of the phase difference due to refractive index mismatch in equation (6). Similar aberration measurements in a gel sample were demonstrated in (26), also showing a linear dependence of spherical aberration on depth.

Measurement of PSFs in tissue samples

Next, we wanted to investigate aberrations in biological tissue samples. To this end, we measured PSFs of the beads injected into the fixed mouse brain tissue. In addition, we investigated the PSFs of small (≤100 nm) spherical agglomerates of Caveolin-1 labelled with Alexa Fluor 647 in similar brain tissue sample. All measurements were performed in layer I (LI) of visual cortex. The results are shown in Fig. S6*J-L* and D-F and Fig. 3. Noticeably, the PSFs in the tissue samples are more elongated along the optical axis and the zero of the z-doughnut PSF is filled up more in the depth comparing to the ones in the agarose-sucrose sample. This can be accounted for by the non-negligible absorption and scattering in the tissue. The influence of absorption and scattering of the tissue on the light intensity (I) can be described by the Lambert-Beer law $I = I_0\, e^{-\mu\, x}$ with the extinction coefficient $\mu = \mu_a + \mu_s$ composed of the absorption and scattering coefficients and x being the optical path length in the tissue. $1/\mu$ then describes the mean free path length in tissue. From attenuation measurements in acute swine brain slices (grey matter) (27), a mean free path length of ~63 µm could be determined. This value may however not be exactly the same for our fixed mouse brain tissue slices. As the optical path length in the tissue is different for rays starting at different positions in the objective back aperture, the outer rays are far more attenuated than the central ones, explaining qualitatively the observed elongation along the optical axis in the depth of the tissue as well as the filling up of the z-doughnut zero.

Still, like in the agarose-sucrose sample, the zero of the vortex PSF is quite robust in the depth, which is most important for MINFLUX localizations. The elongation of the PSF along the optical axis may, however, increases background from the emitters located in different z-planes. As shown in (25), spherical aberrations do not induce bias in 2D MINFLUX, even if the actual focal intensity distribution is not measured and accounted for in the position estimation.

For minimizing the effects of the phase difference onto the PSF, good refractive index matching of the sample to the objective and immersion medium is crucial. Due to the high amount of absorption and scattering in tissue, it is not possible to directly measure the tissue refractive index with the refractometer. We can however deduce the refractive index from the spherical aberration measurements using equation (13). The resulting refractive indices calculated for the tissue samples are shown in Fig. S6*M*. The mean value of calculated refractive index of tissue is about ~1.377, which is, within error, in agreement with OCT measurements of the refractive index in living brain tissue of rats (28) and mice (29).

As shown in Fig. S6*M*, matching the refractive indices of immersion oil and sample minimizes the gradient of the primary spherical aberration with depth. Compared to water ($n_1 = 1.33$), glycerol ($n_1 = 1.456$) and Leica type F immersion oil ($n_1 = 1.518$), silicone immersion oil with $n_1 = 1.406$ best matches the refractive index of tissue. Mixtures of immersion liquid like glycerol and water would match the refractive index of tissue better. However, the high evaporative rates of the components of such mixtures lead to refractive index changes over time, making them less advantageous for MINFLUX nanoscopy, because the duration of MINFLUX acquisitions can be up to hours. As the silicone oil is resistant to evaporation (30), helps to mitigate refractive index mismatch and reduces spherical aberrations, we decided to use a silicone oil immersion objective for MINFLUX nanoscopy in biological tissue.

The problem of absorption and scattering of tissue could in principle be reduced by clearing of the tissue, but this has the disadvantage of more complicated sample preparation and possible sample preparation artifacts.

We reason, based on our study of the focal intensity distribution in tissue, that 2D MINFLUX relying on the vortex excitation beam should be possible up to ~80 µm deep in tissue even without active aberration correction when using a silicone oil objective to match the tissue refractive index.

SI References

1. J. Guy *et al.*, Intracortical Network Effects Preserve Thalamocortical Input Efficacy in a Cortex Without Layers. *Cereb. Cortex* **27**, 4851-4866 (2017).
2. J.-M. Masch *et al.*, Robust nanoscopy of a synaptic protein in living mice by organic-fluorophore labeling. *Proc. Natl. Acad. Sci. U.S.A.* **115**, E8047-E8056 (2018).
3. K. I. Willig *et al.*, Nanoscopy of Filamentous Actin in Cortical Dendrites of a Living Mouse. *Biophys. J.* **106**, L01-L03 (2014).
4. E. Fujiwara, T. D. Cabral, M. Sato, H. Oku, C. M. B. Cordeiro, Agarose-based structured optical fibre. *Sci. Rep.* **10**, 7035 (2020).
5. M. J. Broadhead *et al.*, PSD95 nanoclusters are postsynaptic building blocks in hippocampus circuits. *Sci. Rep.* **6**, 24626 (2016).
6. S. Wakayama *et al.*, Chemical labelling for visualizing native AMPA receptors in live neurons. *Nat. Commun.* **8**, 14850 (2017).
7. I. Hamachi, S. Kiyonaka, S. Wakayama (2015) Development of ligand screening system for neurotransmitter receptors. (Google Patents).
8. F. Balzarotti *et al.*, Nanometer resolution imaging and tracking of fluorescent molecules with minimal photon fluxes. *Science* **355**, 606-612 (2017).
9. K. C. Gwosch *et al.*, MINFLUX nanoscopy delivers 3D multicolor nanometer resolution in cells. *Nat. Methods* **17**, 217–224 (2020).
10. W. Q. Ong, Y. R. Citron, J. Schnitzbauer, D. Kamiyama, B. Huang, Heavy water: a simple solution to increasing the brightness of fluorescent proteins in super-resolution imaging. *Chem. Commun.* **51**, 13451-13453 (2015).
11. S. M. K. Heris (2015) DBSCAN. (Copyright (c) 2015, Yarpiz), p Implementation of DBSCAN Clustering in MATLAB.
12. M. Ester, H.-P. Kriegel, J. Sander, X. Xu (1996) A density-based algorithm for discovering clusters in large spatial databases with noise. in *kdd*, pp 226-231.
13. MATLAB (2011-2017) VISCIRCLES. (The MathWorks, Inc.), p Create circle.
14. C. d. Boor (90) CSCVN `Natural' or periodic interpolating cubic spline curve. (The MathWorks, Inc.).
15. E. T. Y. Lee, Choosing nodes in parametric curve interpolation. *Computer-Aided Design* **21**, 363-370 (1989).
16. B. Luong (2024) N-dimensional histogram. (MATLAB Central File Exchange).
17. MATLAB (1984-2017) ISOSURFACE. (The MathWorks, Inc.), p Isosurface extractor.
18. MATLAB (2009) KNNSEARCH. (The MathWorks, Inc.), p Find K nearest neighbors.
19. J. H. Friedman, J. L. Bentley, R. A. Finkel, An algorithm for finding best matches in logarithmic expected time. *ACM Trans. Math. Softw.* **3**, 209-226 (1977).
20. MATLAB (2013-2015) alphaShape. (The MathWorks, Inc.), p Alpha Shape in 2D and 3D.
21. A. I. Sobolevsky, M. P. Rosconi, E. Gouaux, X-ray structure, symmetry and mechanism of an AMPA-subtype glutamate receptor. *Nature* **462**, 745-756 (2009).
22. K. H. Hanisch, D. Stoyan, Formulas for the second-order analysis of marked point processes. *Series Statistics* **10**, 555-560 (1979).
23. N. Rebola *et al.*, Distinct nanoscale calcium channel and synaptic vesicle topographies contribute to the diversity of synaptic function. *Neuron* **104**, 693-710 (2019).
24. F. Zernike, Beugungstheorie des Schneidenverfahrens und seiner verbesserten Form, der Phasenkontrastmethode. *Physica* **1**, 689-704 (1934).
25. G.-m. Dai, Scaling Zernike expansion coefficients to smaller pupil sizes: a simpler formula. *J. Opt. Soc. Am. A* **23**, 539-543 (2006).
26. S. Bancelin, L. Mercier, E. Murana, U. V. Nägerl, Aberration correction in stimulated emission depletion microscopy to increase imaging depth in living brain tissue. *Neurophotonics* **8**, 035001 (2021).
27. A. Taddeucci, F. Martelli, M. Barilli, M. Ferrari, G. Zaccanti, Optical properties of brain tissue. *J. Biomed. Opt.* **1** (1996).
28. J. Sun, S. J. Lee, L. Wu, M. Sarntinoranont, H. Xie, Refractive index measurement of acute rat brain tissue slices using optical coherence tomography. *Opt. Express* **20**, 1084-1095 (2012).
29. A. J. Lee *et al.*, Volumetric Refractive Index Measurement and Quantitative Density Analysis of Mouse Brain Tissue with Sub-Micrometer Spatial Resolution. *Adv. Photonics Res.* **4**, 2300112 (2023).
30. A. Samuelsson, Silicone Immersion Objectives Answer the Call for Higher Resolution. *Photonics spectra* **51** (2017).

Figures S1 to S17

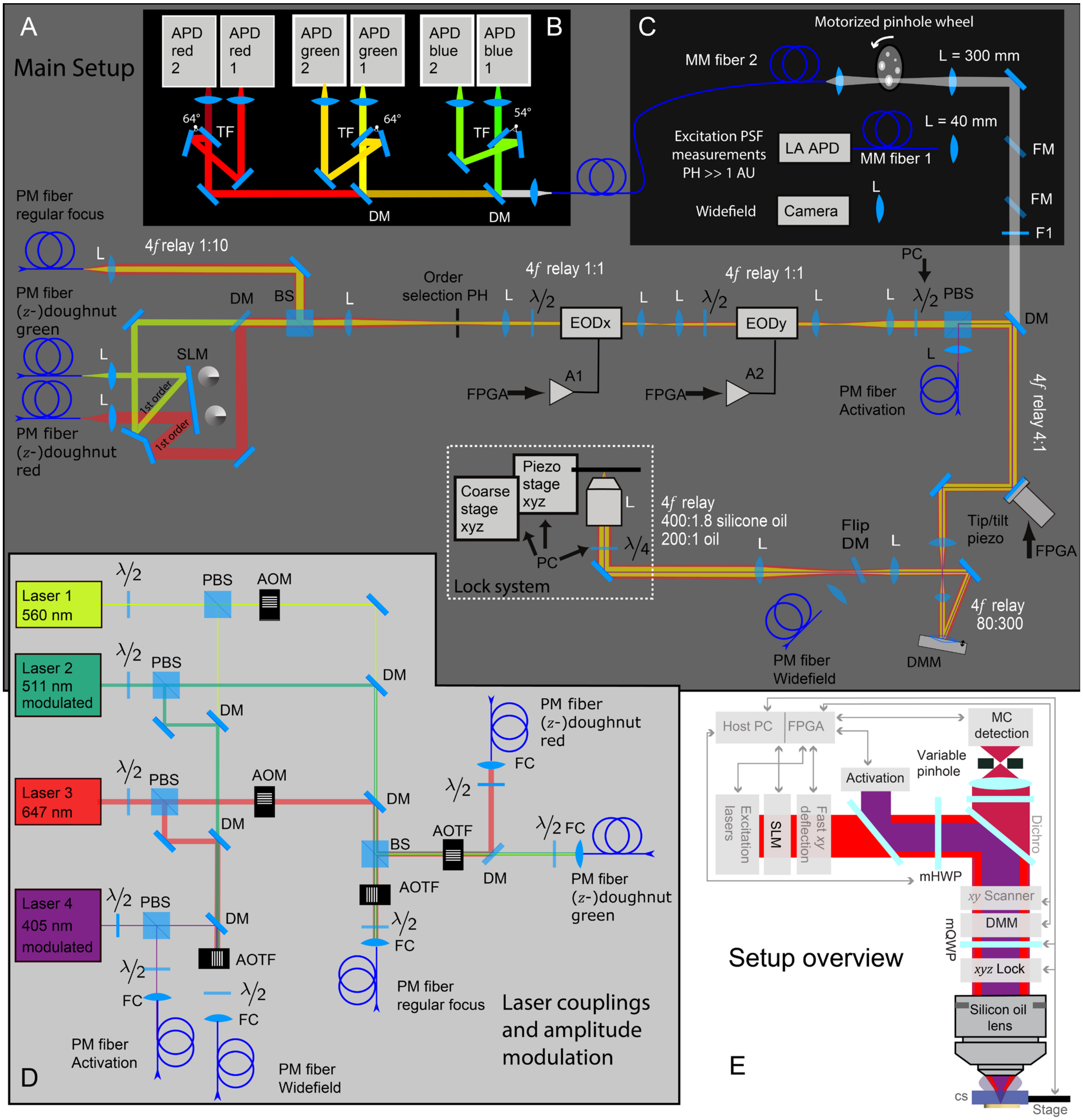

Fig. S1: Overview of MINFLUX setup. Detailed drawing of setup with all crucial optical components (not to scale). (*A*) Main setup consisting of the excitation, activation and detection beam paths, phase modulation, scanner system, microscope stage and objective lens. Depth adaptable lock system shown in detail in Fig. 2. (*B*) Light-proof multicolor detection box for spectrally separated confocal detection. (*C*) Detection box with the camera for widefield imaging, the Large Area (LA) APD for PSF measurements and the variable confocal pinhole (widefield camera and LA APD not illuminated). (*D*) Excitation and activation laser box with power modulator. Boxes are linked by optical fibers for better stability and exterior light protection. (*E*) MINFLUX setup overview from Fig. 1*A*, shown again for orientation. Abbreviations for components are explained in Table S10.

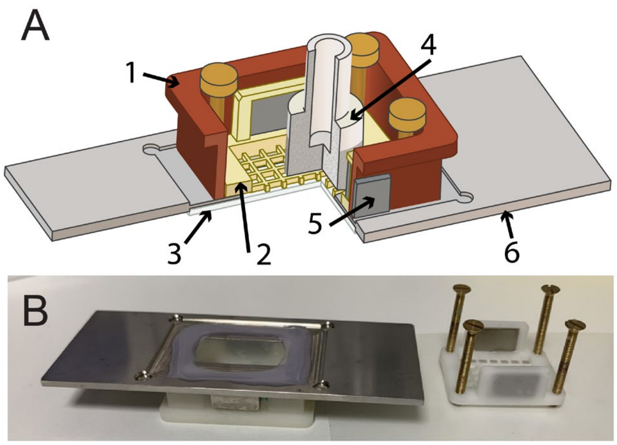

Fig. S2: Chamber for living slice imaging. (*A*) Schematic drawing of chamber with oxygen supply: **1**-basin, **2**-grid, **3**-coverslip, **4**-percolator delivering gas, **5**-magnet, **6**-steel slide. (*B*) Life imaging chamber mounted on slide (left) and the grid for fixation of the tissue slice (right).

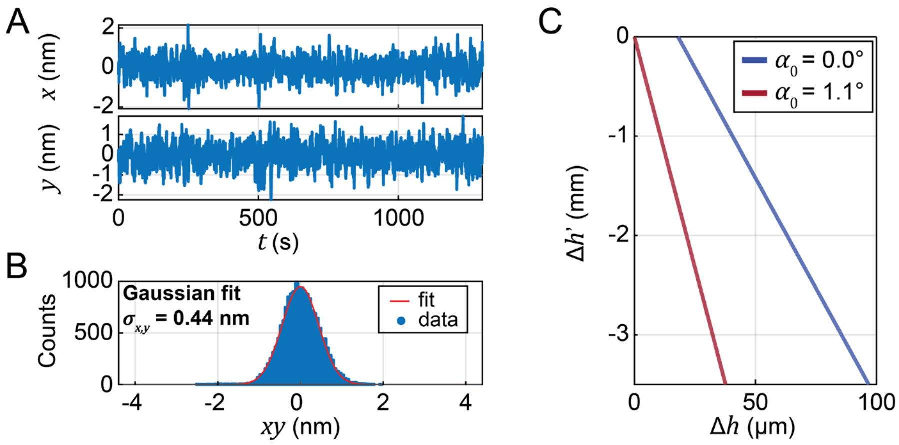

Fig. S3: Depth-adaptable focus lock system. (*A*) Example time traces of the focus lock position deviations in x and y for a stability measurement directly on the coverslip. Measurement was performed in an agarose-sucrose gel with refractive index of 1.38. (*B*) Histogram of combined x and y focus lock position deviations. Gaussian fit to the data shown in red. The resulting focus lock precision for a measurement of ~10 min is 0.44 nm. (*C*) Simulation of beam movement $\Delta h'$ on camera due to sample movement Δh along the optical axis for different angles α_0 of the incoming beam that can be selected by the absolute positionable mirror. σ_{xy}: position stability.

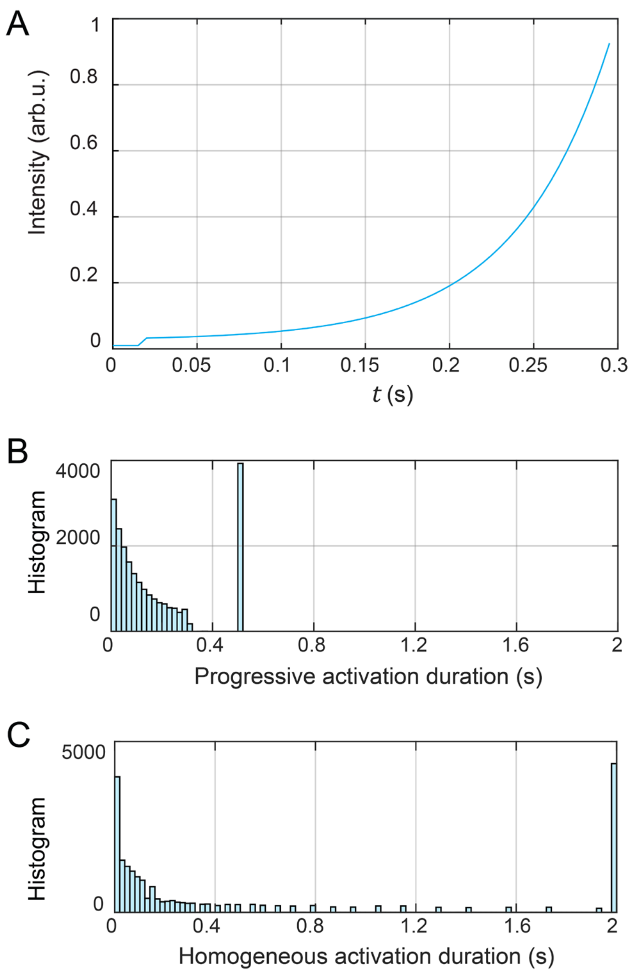

Fig. S4: Progressive Activation. (*A*) Temporal change of activation intensity. (*B*) Histogram of recorded activation durations during acquisition of an image. The large peak at 0.5 s contains the instances in which no activation of a molecule has taken place (including the time for the piezo mirror jump of 200 ms). (*C*) Estimated histogram of activation durations, based on the doses per activation during the image acquisition expected for a homogeneous activation intensity of 2 % of the maximum intensity.

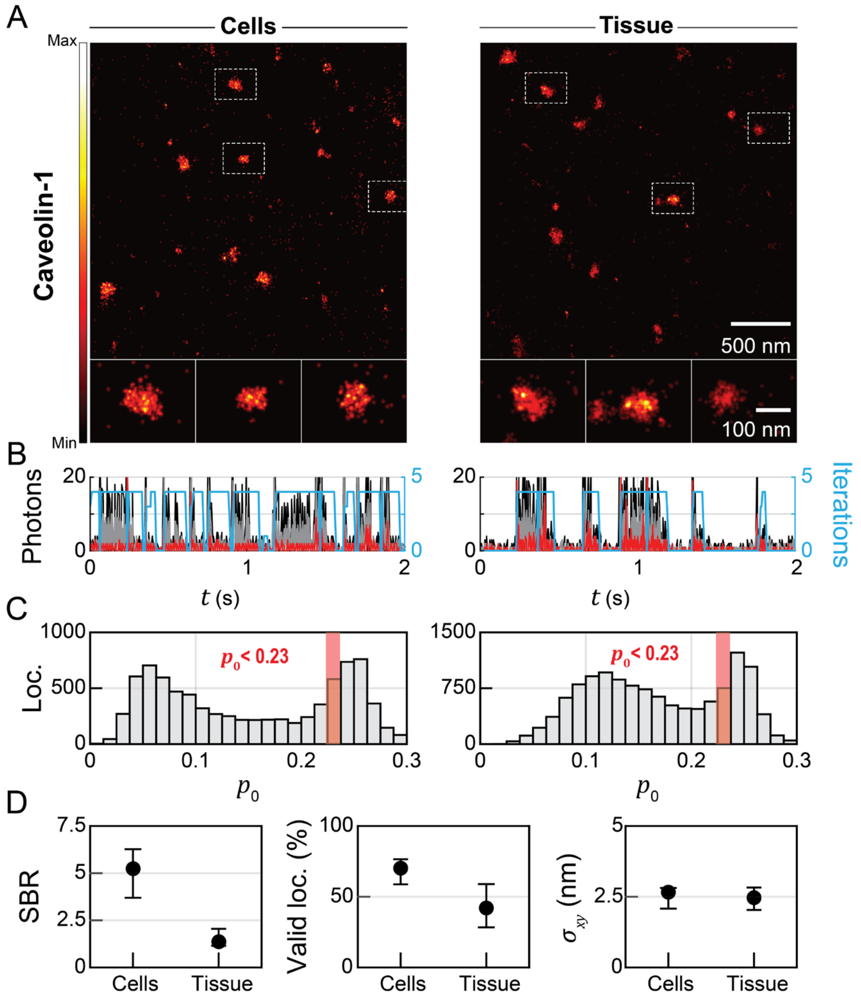

Fig. S5: MINFLUX imaging of Caveolin-1 in cells and tissue. (*A*) Overview MINFLUX images of Caveolin-1 labeled with primary and secondary antibody by Alexa Fluor 647 in cultured U-2 OS cells and mouse brain tissue close to the coverslip (upper panel). The lower panels show enlarged views of three selected Caveolin-1 clusters found in dotted white boxes indicated in the overviews. (*B*) Selected photon trace showing molecule emission events and iterative centering of the targeted-coordinate pattern onto the molecules for the measurements shown above. (*C*) Histograms of p_0-values of localizations in the images. (*D*) Median values and interquartile range of SBR, ratio of valid localizations and localization precision calculated for MINFLUX images of Caveolin-1 in cells (6 images, 22 478 664 localizations) and tissue (23 images, 703 924 localizations). Loc.: localizations; SBR: signal-to-background ratio; σ_{xy}: localization precision.

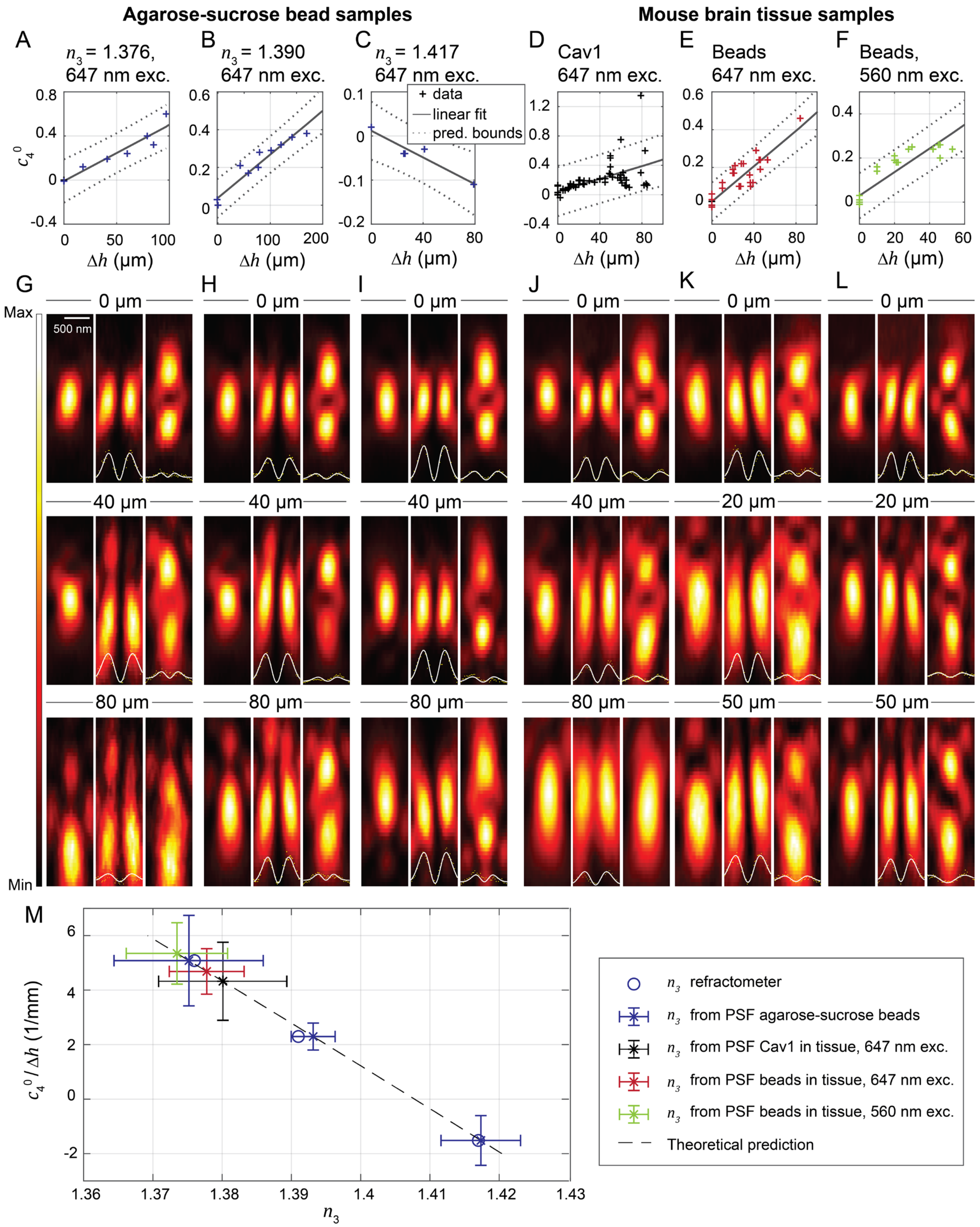
Agarose-sucrose bead samples
Mouse brain tissue samples
A
n_3 = 1.376, 647 nm exc.
B
n_3 = 1.390 647 nm exc.
C
n_3 = 1.417 647 nm exc.
+ data
linear fit
pred. bounds
D
Cav1 647 nm exc.
E
Beads 647 nm exc.
F
Beads, 560 nm exc.
c_4^0
Δh (μm)
G
H
I
J
K
L
Max
Min
500 nm
0 μm
40 μm
80 μm
20 μm
50 μm
M
c_4^0 / Δh (1/mm)
n_3
n_3 refractometer
n_3 from PSF agarose-sucrose beads
n_3 from PSF Cav1 in tissue, 647 nm exc.
n_3 from PSF beads in tissue, 647 nm exc.
n_3 from PSF beads in tissue, 560 nm exc.
Theoretical prediction

Fig. S6: Focal intensity distributions (PSFs) in different imaging depths. Spherical aberration vs. depth measured in agarose-sucrose samples (*A*-*C*) and in mouse brain tissue sample (*D*-*F*) for 647 nm or 560 nm excitation wavelength. (*G*-*L*) Axial cuts through examples of PSFs for three different imaging depths for the corresponding sample type. Imaging depths were rounded to 10 µm. Regular focus, doughnut and z-doughnut PSFs are shown. Line profiles perpendicular to the optical axis characterizing the zero are shown together with a doughnut shaped fit for each doughnut PSF and z-doughnut PSF. While the zero contrast of the doughnut is still good in the depth, the zero contrast of the z-doughnut decreases rapidly. (*M*) Data summary showing the relation between the depth-dependent spherical aberration and the refractive index of the sample together with the result of the refractometer measurements of the agarose-sucrose gels. Cav1: Caveolin-1; Δh: depth; n_3: refractive index; exc.: excitation; pred.: prediction.

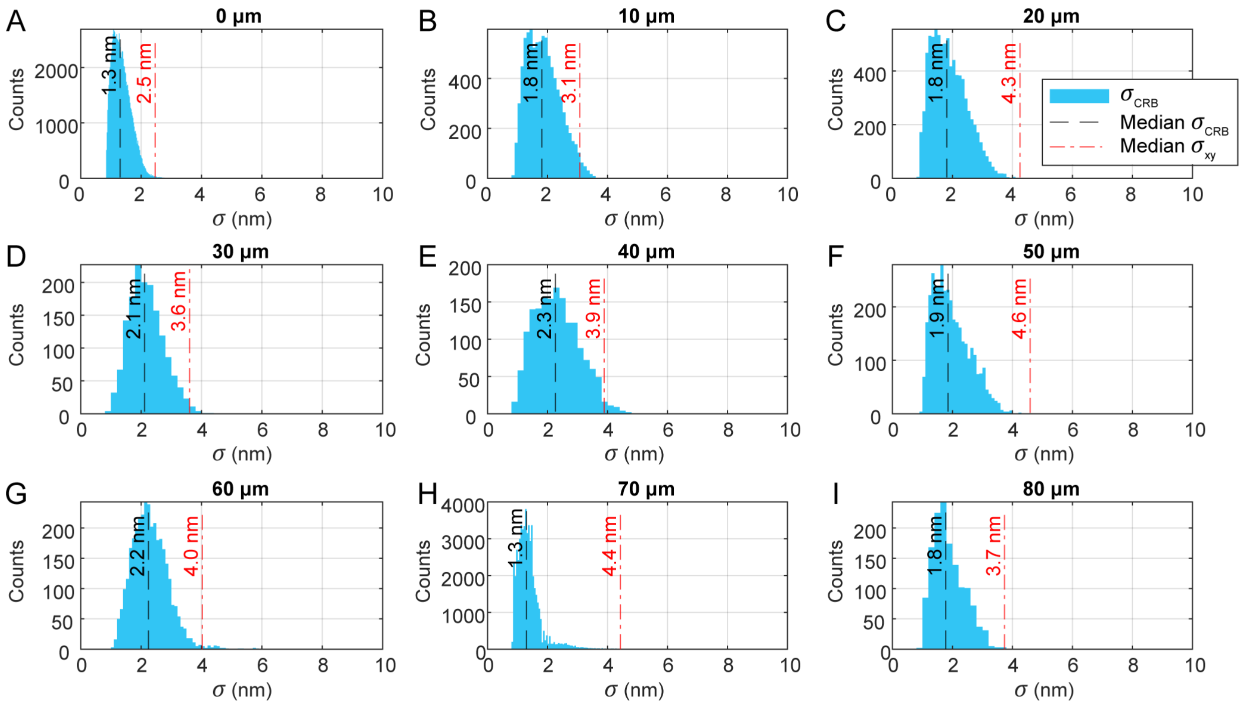

Fig. S7: MINFLUX imaging of Caveolin-1 in different depths of mouse brain tissue. (*A*) Histogram and median value of Cramér-Rao-bounds (σ_{CRB}) calculated from experimentally determined signal-to-background ratios (SBR) per localization compared to the median measured localization precision (σ_{xy}) from all Caveolin-1 images acquired 0 µm deep in tissue. (*B*-*I*) Same analysis for images acquired 10-80 µm deep in tissue. σ - localization precision.

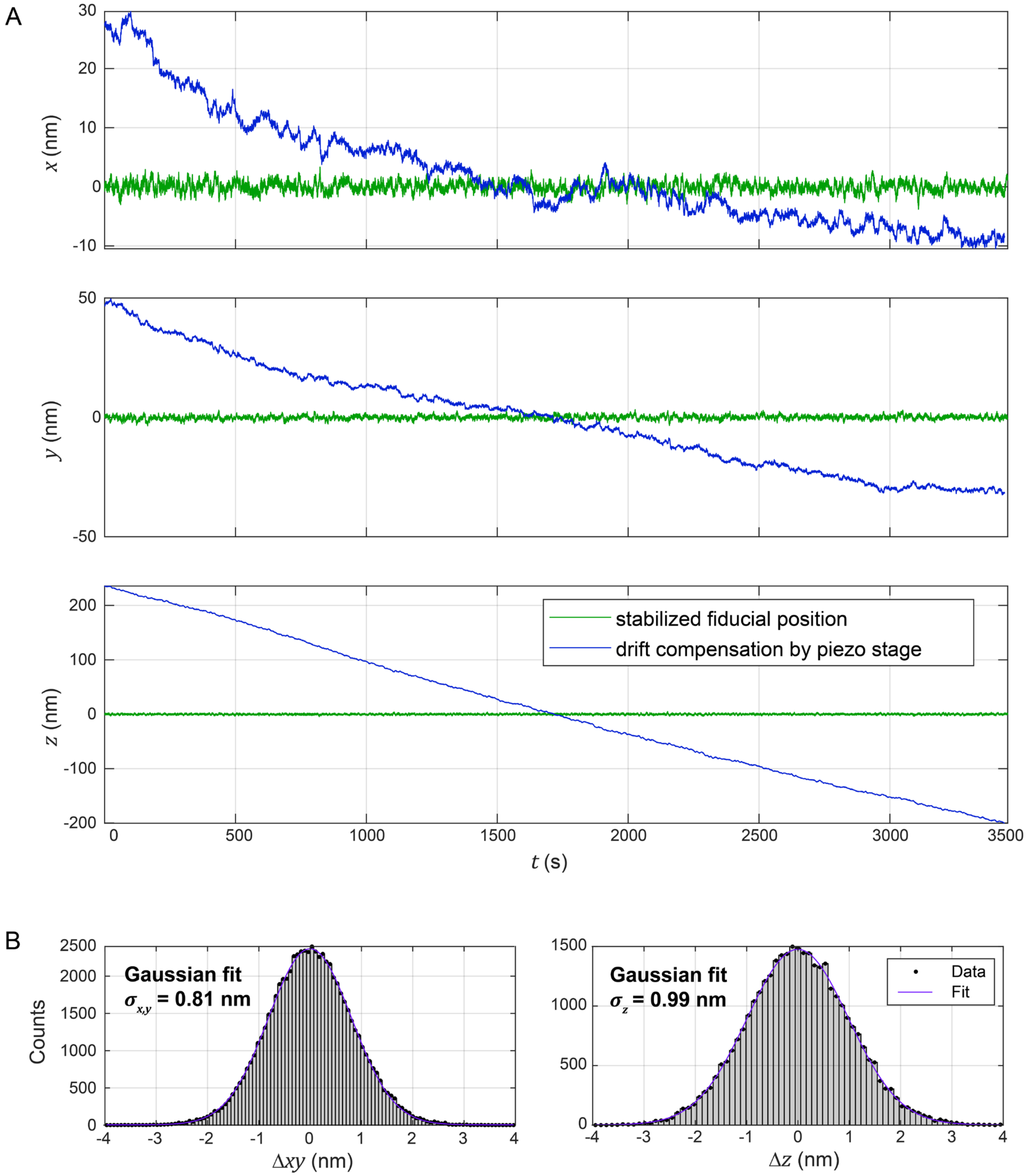

Fig. S8: 3D focus lock stability during measurement in tissue close to the coverslip. (*A*) Time trace of fiducial position in x, y, and z respectively, plotted together with the drift compensation movement of the fine piezo stage. (*B*) Histograms (lateral and axial) of focus lock deviation from set position. The stability measurement corresponds to the MINFLUX image shown in Fig. 4*A*, 0 µm depth.

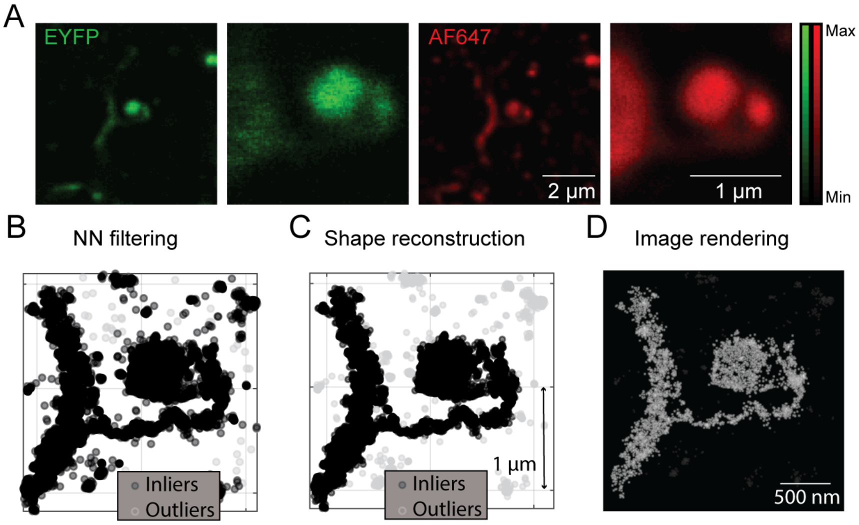

Fig. S9: Delineation of actin in dendritic spines. (*A*) Confocal overview images and zoom-ins to the region of interest shown for EYFP and Alexa Fluor 647 channel. (*B*) Raw MINFLUX localizations from the ROI with nearest neighbor filtering (NN filtering), shape reconstruction (*C*) and image rendering as sum of Gaussian distributions (*D*).

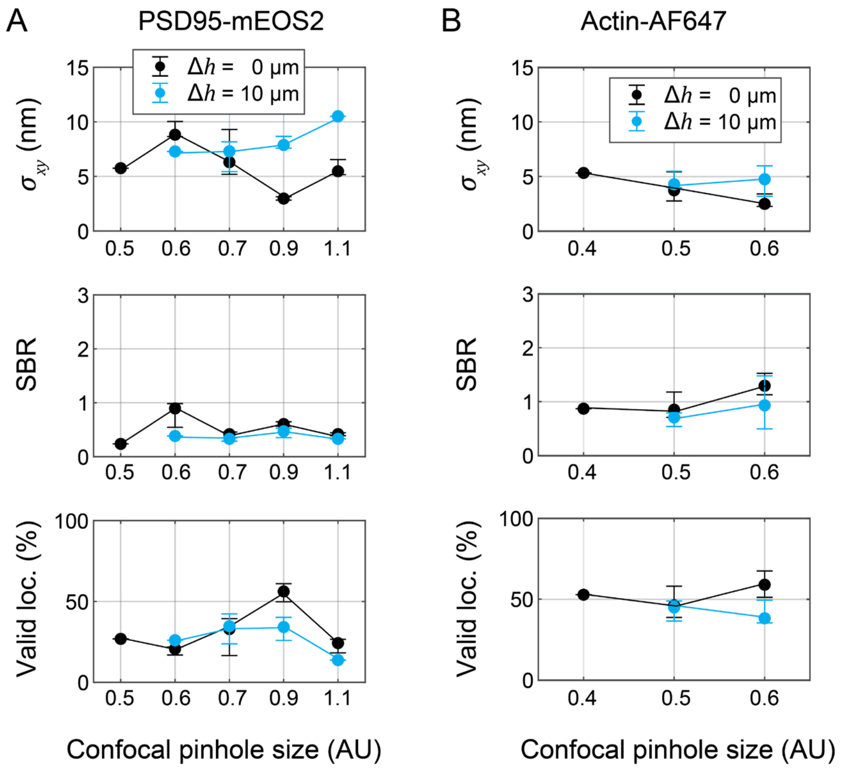

Fig. S10: Influence of confocal pinhole size on the MINFLUX metrics. Localization precision, SBR and percentage of valid localizations were evaluated for different confocal pinhole sizes directly on the cover slip (0 µm) and at ~10 µm depth for 2 different samples; (*A*) PSD95-mEos2 and (*B*) Actin-Alexa Fluor 647. Median values with interquartile range are plotted. Sample size for PSD95 with 0.5 AU: 1 image, 23 729 localizations, PSD95 with 0.6 AU: 4 images, 2 071 616 localizations, PSD95 with 0.7 AU: 42 images, 1 847 111 localizations:PSD95 with 0.9 AU: 27 images, 335 680 localizations, PSD95 with 1.1 AU: 8 images, 448 405 localizations. Alexa Fluor 647 with 0.4 AU: 1 image, 5 765 localizations, Alexa Fluor 647 with 0.5 AU: 64 images, 37 515 021 localizations, Alexa Fluor 647 with 0.6 AU: 24 images, 16 798 087 localizations. Δh: depth; AF647: Alexa Fluor 647; loc.: localizations; SBR: signal to background ratio; σ_{xy}: localization precision.

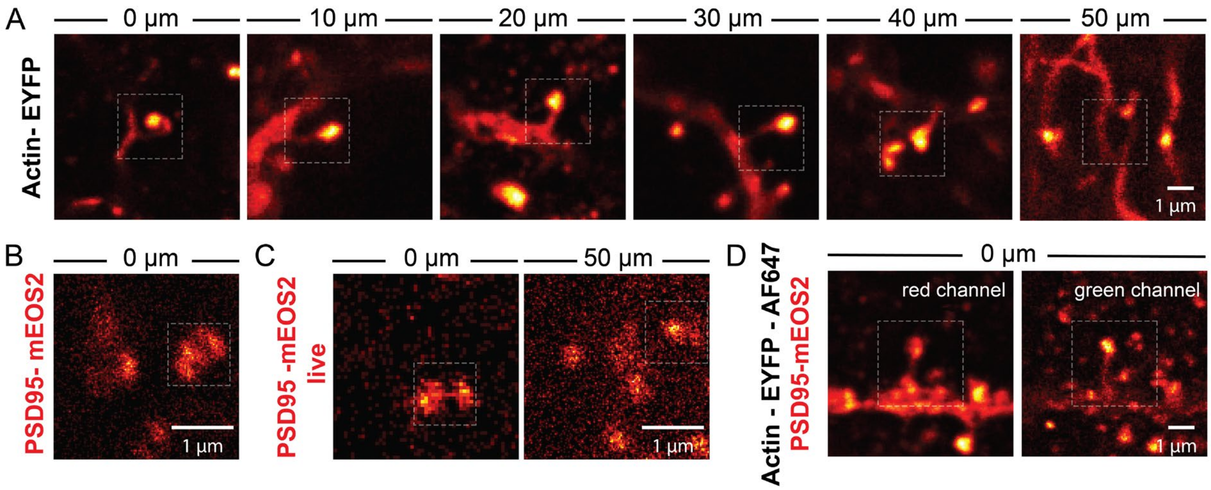

Fig. S11: Confocal overview images for the MINFLUX nanoscopy shown in Fig. 4. (*A*) Confocal images of LifeAct-EYFP with excitation at 511 nm (green channel) in all imaging depths. Regions selected for MINFLUX nanoscopy indicated by white dashed square. (*B*) Confocal image of PSD95-mEos2 acquired before photoconversion of mEos2 in the green channel. (*C*) Confocal images of PSD95-mEos2 in living tissue in 0 µm and 50 µm imaging depth acquired in the green channel. (*D*) Two-color confocal image shown separately for both color channels. Red channel showing Alexa Fluor 647 labeling of EYFP, green color channel showing EYFP and mEos2.

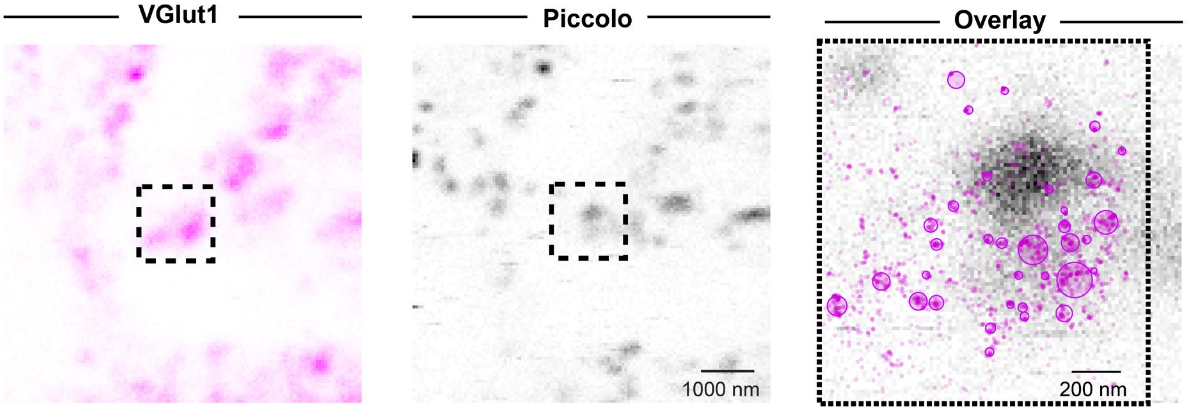

Fig. S12: Confocal overview images of VGlut1 and Piccolo distribution and smaller inset overlayed with MINFLUX acquisitions of VGlut1. Confocal overview of VGlut1 (left) and Piccolo (center) distributions. (Right) Smaller region of confocal image overlayed with MINFLUX image (black outline).

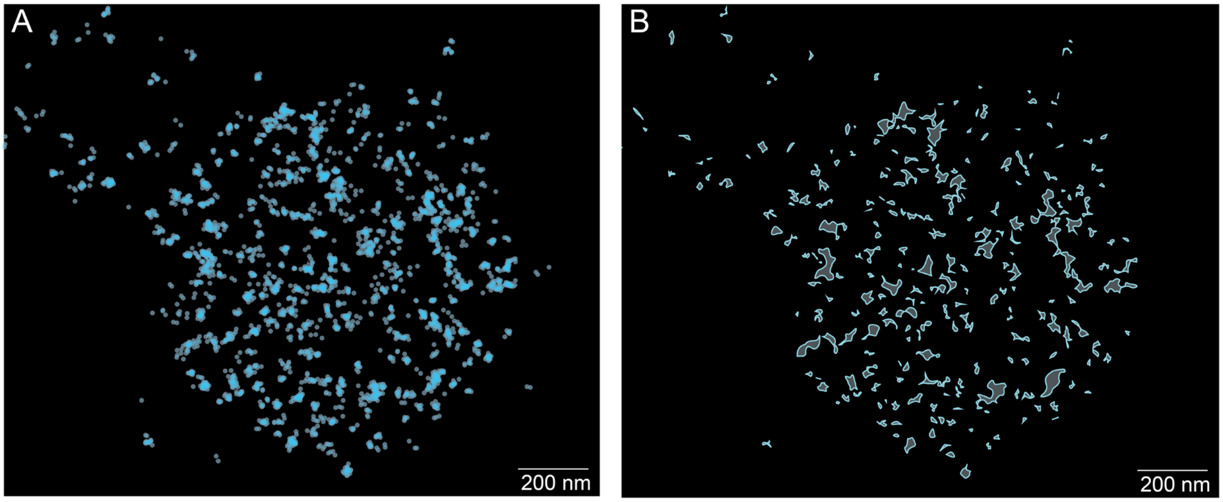

Fig. S13: AMPAR clusters shown in Fig. 5*D*,*E*. (*A*) Raw AMPAR label-site positions. (*B*) *Dbscan*-clustering of AMPAR label-site positions.

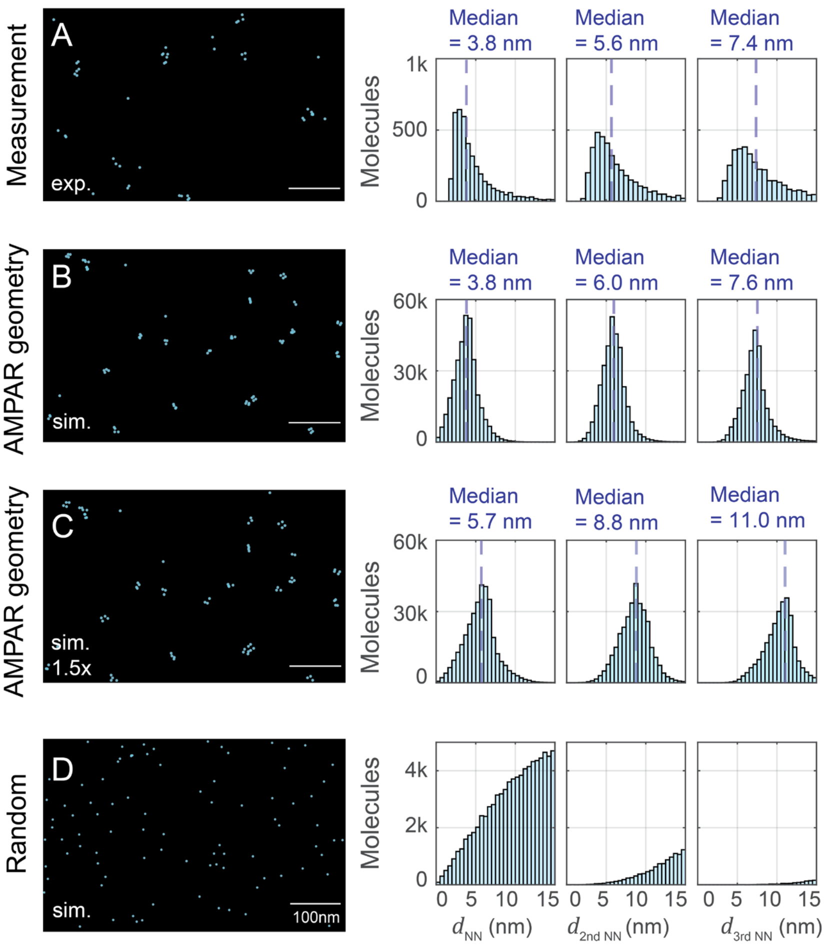

Fig. S14: Comparison of AMPAR measurement with simulations. (*A*) Selected field of view of 2D MINFLUX image. On the right, the nearest neighbor distances, the second nearest neighbor distances and the third nearest neighbor distances between AMPARs subunit localizations are shown. (*B*) Selected field of view of simulation of AMPARs relying on the label position geometry extracted from the protein data base file 3KG2. Nearest neighbor distances between simulated subunits, accumulated for 100 simulated images. Simulated fraction of AMPARs with 4 labeled subunits: 2/3; 3 labeled subunits: 1/6; 2 labeled subunits: 1/12; 1 labeled subunit: 1/12. (*C*) Same simulation as before, with the adaptation that the AMPAR label position geometry is scaled by a factor of 1.5. (*D*) Simulation with same density of molecules as before, but randomly distributed. Exp.: experimental data, sim.: simulated data, sim. 1.5x: simulation with 1.5x scaled base geometry, NN: nearest neighbor.

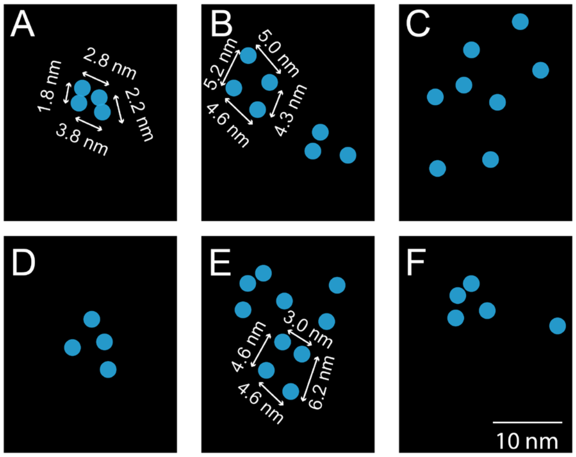

Fig. S15: AMPAR geometries overview. Selected ROIs (*A-F*) from the AMPAR image shown in Fig. 5*D*. Some distances between labels that are assumed to be within one receptor are indicated.

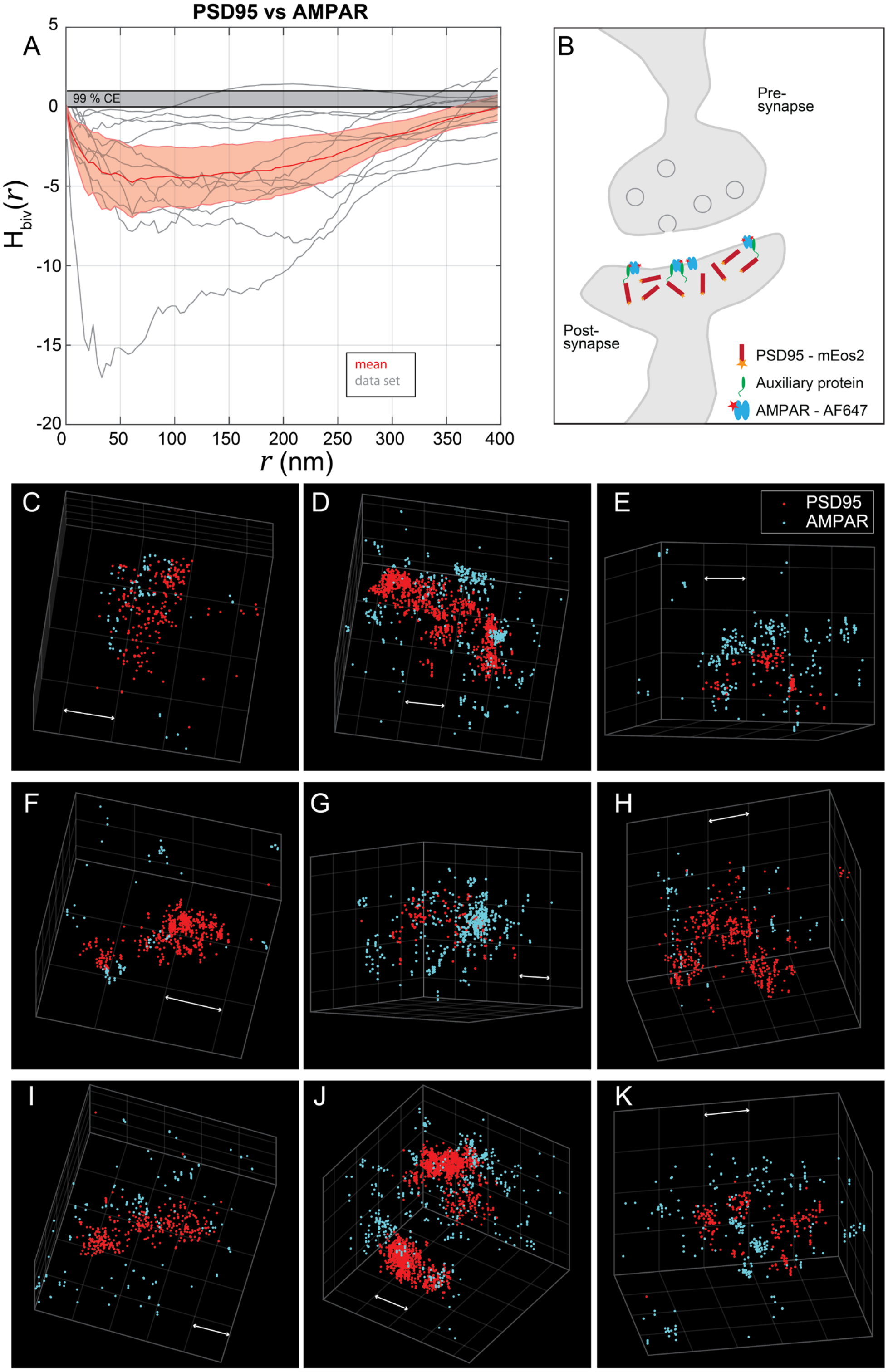

Fig. S16: Relative arrangement of PSD95 and AMPAR in 3D two-color MINFLUX measurements. (*A*) Bivariate Ripley´s analysis of the relative arrangement of PSD95 and AMPAR for 3D two-color measurements. (*B*) Schematic model of AMPAR and PSD95 with their labels. (*C*-*K*) Example volumes. TARP: transmembrane AMPA receptor regulatory proteins. Scale bars are 200 nm.

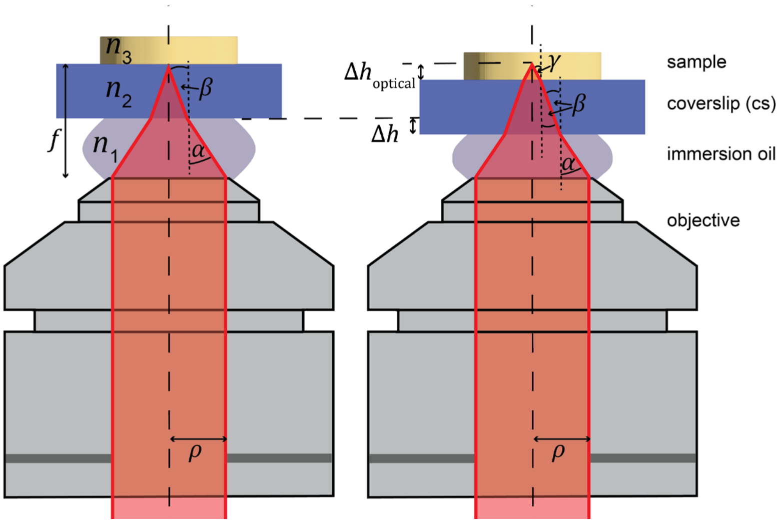

Fig. S17: Schematic illustration of focusing into a sample with refractive index mismatch to the immersion oil and the coverslip. Refractive indices $(n_1 - n_3)$ of immersion oil, glass coverslip and sample, focal length f, angles of incidence (α, β, γ), radius of the objective back aperture ρ and imaging depth Δh are indicated.

Tables S1 to S10

Table S1: Localization patterns for 2D MINFLUX adapted from (9).

**Excitation wavelength (nm)**	**Localization pattern**					
**560**	Iteration Index	Beam shape	TCP	L (nm)	Max. #Phot. to collect	Est. param.
	1	Regular focus	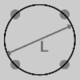	300	100	0.8
	2	Regular focus	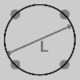	300	100	0.8
	3	Doughnut	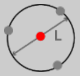	150	100	0.883, 6.623
	4	Doughnut	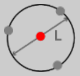	100	250	0.728, 9.15
	5	Doughnut	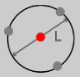	100	10 000	0.729, 9.114
**647**	Iteration Index	Beam shape	TCP	L (nm)	Max. #Phot. to collect	Est. param.
	1	Regular focus	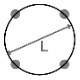	300	100	0.8
	2	Regular focus	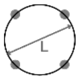	300	100	0.8
	3	Doughnut	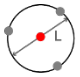	150	100	0.875, 6.857
	4	Doughnut	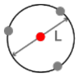	100	250	0.719, 9.305
	5	Doughnut	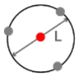	100	10 000	0.719, 9.269

Table S2: 2D MINFLUX measurement parameters for different samples. Laser powers are measured close to the objective. Localization patterns are detailed in Table S1.

Sample	U-2 OS cells antiCaveolin-1-antiAlexa Fluor 647	Tissue antiCaveolin-1-antiAlexa Fluor 647	Tissue LifeAct -EYFP antiGFP -antiAlexa Fluor 647	Tissue AMPAR -CAM2 Alexa Fluor 647	Tissue PSD95-mEos2
**Excitation wavelength (nm)**	647	647	647	647	560
**Laser power for excitation with doughnut focus (μW)**	100	100	100	100	14-40
**Laser power for excitation with regular focus (μW)**	60	60-80	60	60	8-25
**Laser power for activation @ 405 nm (μW)**	Progressive activation up to 1	Progressive activation up to 0.1- 1.4	Progressive activation up to 1	Progressive activation up to 0.1	0.006-1.1
**Confocal pinhole diameter (AU)**	0.62	0.46 -0.54	0.46 - 0.62	0.62	0.53 - 0.9
**Pixel pitch (distance between mosaic positions at which we activate) (nm)**	200	200	200	200	150-200
**Maximum activation time at mosaic index (s)**	1.2	0.3-1.2	0.3-1.2	0.6	0.3-1.2
**Reset of activation time with molecule**	Yes	Yes	Yes	Yes	Yes
**Maximum time at mosaic position (s)**	10	10-20	10	10	10-15
**Localization pattern**	2D MINFLUX 647 nm excitation	2D MINFLUX 647 nm excitation	2D MINFLUX 647 nm excitation	2D MINFLUX 647 nm excitation	2D MINFLUX 560 nm excitation

Table S3: 3D MINFLUX localization schemes for z-doughnut vs. doughnut-regular focus comparison measurements and 3D two-color measurements. z-doughnut localization scheme adapted from (9).

Main excitation beam(s)	Localization pattern						
	Iter. Ind.	Beam shape	TCP	# exposures	L (nm)	Max. #Phot. to collect	Est. param.
**Doughnut 647 nm + regular focus 647 nm**	1	regular focus	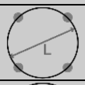	4	300	100	0.8
	2	Regular focus	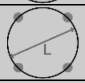	4	300	100	0.8
	3	Regular focus	1D, z	2	1200	100	0.15
	4	Regular focus	1D, z	2	1200	100	0.15
	5	Regular focus	1D, z	2	1200	100	0.15
	6	Doughnut	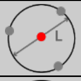	4	150	100	0.89, 7.18
	7	Doughnut	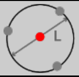	4	100	200	0.57, 10.8
	8	Doughnut / regular focus	3D	6	100, 750	10 000	0.57, 10.8, 0.35
	Iter. Ind.	Beam shape	TCP	# exposures	L (nm)	Max. #Phot. to collect	Est. param.
**z-doughnut 647nm**	1	Regular focus	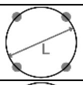	4	300	100	0.8
	2	Regular focus	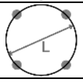	4	300	100	0.8
	3	Regular focus	1D, z	2	1200	100	0.15
	4	z-doughnut	1D, z	2	300	100	0.55
	5	z-doughnut	1D, z	2	200	150	0.55
	6	z-doughnut	3D	7	150	150	0.88, 23.5
	7	z-doughnut	3D	7	100	200	0.58, 31.5
	8	z-doughnut	3D	7	100	10 000	0.58, 31.3

**Two-color measurements with doughnut for 560nm**	Iter. Ind.	Beam shape	TCP	# exposures	L (nm)	Max. #Phot. to collect	Est. param.
	1	Regular focus	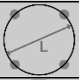	4	300	100	0.8
	2	Regular focus	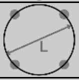	4	300	100	0.8
	3	Regular focus	1D, z	2	1200	100	0.5
	4	Regular focus	1D, z	2	1200	100	0.5
	5	Regular focus	1D, z	2	1200	100	0.5
	6	Doughnut	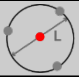	4	150	100	0.89, 7.18
	7	Doughnut	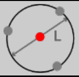	4	100	200	0.57, 10.8
	8	Doughnut / regular focus	3D	6	100, 750	10 000	0.57, 10.8, 0.35

**Two-color measurements with doughnut for 647nm**	Iter. Ind.	Beam shape	TCP	# exposures	L (nm)	Max. #Phot. to collect	Est. param.
	1	Regular focus	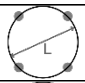	4	300	100	0.8
	2	Regular focus	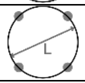	4	300	100	0.8
	3	Regular focus	1D, z	2	1200	100	0.5
	4	Regular focus	1D, z	2	1200	100	0.5
	5	Regular focus	1D, z	2	1200	100	0.5
	6	Doughnut	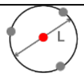	4	150	100	0.89, 7.18
	7	Doughnut	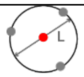	4	100	200	0.57, 10.8
	8	Doughnut / regular focus	3D	6	100, 750	10 000	0.57, 10.8, 0.35

Table S4: 3D MINFLUX acquisition parameters for comparison measurements between z-doughnut and doughnut/regular focus. Localization schemes are detailed in Table S3.

**Sample**	**Tissue LifeAct-EYFP antiGFP - antiAlexa Fluor 647**	**Tissue LifeAct-EYFP antiGFP -antiAlexa Fluor 647**
**Excitaion wavelength (nm)**	647	647
**Laser power for excitation with doughnut focus (μW)**	90	90
**Laser power for excitation with regular focus focus (μW)**	90	90
**Laser power for activation @ 405 nm (μW)**	Progressive activation up to 0.1	Progressive activation up to 0.1
**Confocal pinhole diameter (AU)**	0.6	0.6
**Pixel pitch (distance between mosaic positions at which we activate) (nm)**	200	200
**Maximum activation time at mosaic index (s)**	0.6	0.6
**Reset of activation time with molecule**	yes	yes
**Maximum time at mosaic position (s)**	10	10
**Localization pattern**	Doughnut/regular focus	z-doughnut

Table S5: 3D 2-color MINFLUX acquisition parameters. Localization schemes are detailed in Table S3.

**Sample**	**Tissue PSD95-mEos2 LifeAct-EYFP antiGFP-antiAlexa Fluor 647**	**Tissue PSD95-mEos2 LifeAct -EYFP antiGFP-antiAlexa Fluor 647**	**Tissue mEos2-PSD95 AMPA CAM2-Alexa Fluor 647 (live stained)**	**Tissue mEos2-PSD95 AMPA CAM2-Alexa Fluor 647 (live stained)**
**Excitation wavelength (nm)**	647	560	647	560
**Laser power for excitation with doughnut focus (μW)**	100	25	100	25
**Laser power for excitation with regular focus (μW)**	80	25	80	25
**Laser power for activation @ 405 nm (μW)**	(Progressive activation to) 0.05-0.1	(Progressive activation to) 0.006-0.1	0.1	0.1
**Confocal pinhole diameter (AU)**	0.6	0.6	0.6	0.6
**Pixel pitch (distance between mosaic positions at which we activate) (nm)**	150-200	150	150-200	150
**Maximum activation time at mosaic index (s)**	0.3	0.3	0.3	0.3
**Reset of activation time with molecule**	Yes	Yes	Yes	Yes
**Maximum time at mosaic position (s)**	10	10	10	10
**Localization pattern**	Doughnut/ regular focus	Doughnut/ regular focus	Doughnut/ regular focus	Doughnut/ regular focus

Table S6: Fluorophore-related adaptation of parameters for post processing of MINFLUX traces of the depth imaging series and 2-color images in 2D and 3D. The $p_{0,\max}$ value for filtering of the localizations is set with respect to the “Background peak” center μ_2 of the p_0 histogram. For 2D MINFLUX, the value μ_2 = 0.25.

**Fluorophore**	**Actin Alexa Fluor 647**<br>**Cav1 Alexa Fluor 647**	**mEos2**	**AMPA Alexa Fluor 647 (live stained)**	**mEos2 (in live stained slice)**
**HMM parameters**	t_{On} = 0.5 s<br>t_{Off} = 0.1 s<br>$t_{\mathrm{Blink,\ On}}$ = 0.001 s<br>$t_{\mathrm{Blink,\ Off}}$ = 0.0001 s	t_{On} = 5 s<br>t_{Off} = 1 s<br>$t_{\mathrm{Blink,\ On}}$ = 0.005 s<br>$t_{\mathrm{Blink,\ Off}}$ = 0.0005 s	t_{On} = 0.5 s<br>t_{Off} = 0.1 s<br>$t_{\mathrm{Blink,\ On}}$ = 0.001 s<br>$t_{\mathrm{Blink,\ Off}}$ = 0.0001 s	t_{On} = 5 s<br>t_{Off} = 1 s<br>$t_{\mathrm{Blink,\ On}}$ = 0.005 s<br>$t_{\mathrm{Blink,\ Off}}$ = 0.0005 s
**Molecule threshold for post processing**	Automatic, quantile range [0,0.95]	Automatic, quantile range [0,0.9]	Automatic, quantile range [0,0.95]	Automatic, quantile range [0,0.9]
**Number of photons for re-segmentation**	2000	1000	2000	1000
**Minimum number of photons for localization**	50	100	50	100
$\boldsymbol{p_{0,max}}$	0.92 μ_2	0.92 μ_2	0.8 μ_2	0.83 μ_2

Table S7: Postprocessing parameters for the 2D AMPAR and VGlut1 datasets.

Sample	AMPAR Alexa Fluor 647	VGlut1 Alexa Fluor 647
**HMM parameters**	t_{On} = 0.5 s<br>t_{Off} = 0.1 s<br>$t_{\mathrm{Blink,\ On}}$ = 0.001 s<br>$t_{\mathrm{Blink,\ Off}}$ = 0.0001 s	t_{On} = 0.5 s<br>t_{Off} = 0.1 s<br>$t_{\mathrm{Blink,\ On}}$ = 0.001 s<br>$t_{\mathrm{Blink,\ Off}}$ = 0.0001 s
**Molecule threshold for post-processing (kHz)**	20	20
**Number of photons for re-segmentation**	2000	2000
**Minimum number of photons for localization**	100	100
$\boldsymbol{p_{0,max}}$	0.2 (corresponds to 0.8 μ_2)	0.23 (corresponds to 0.92 μ_2)

Table S8: Number of MINFLUX images and localizations from which the statistics shown in Fig. 4*C-E* are calculated.

Sample	Number of localizations	Number of images
**Tissue, PSD95-mEos2**	10 332 325 (~0 μm)<br>3 374 698 (~10 μm)<br>6 955 906 (~20 μm)<br>1 428 416 (~30 μm)<br>255 912 (~40 μm)<br>10 815 (~50 μm)	55 (~0 μm)<br>29 (~10 μm)<br>10 (~20 μm)<br>5 (~30 μm)<br>8 (~40 μm)<br>1 (~50 μm)
**Living tissue, PS95-mEos2**	4 116 582 (~0 μm)<br>4 380 655 (~10 μm)<br>17 250 (~20 μm)<br>16 625 (~50 μm)	24 (~0 μm)<br>15 (~10 μm)<br>1 (~20 μm)<br>1 (~50 μm)
**Tissue, actin-Alexa Fluor 647**	108 281 545 (~0 μm)<br>24 485 604 (~10 μm)<br>2 062 425 (~20 μm)<br>3 786 615 (~30 μm)<br>4 158 935 (~40 μm)<br>6 354 430 (≳ 50 μm)	38 (~0 μm)<br>30 (~10 μm)<br>7 (~20 μm)<br>6 (~30 μm)<br>7 (~40 μm)<br>3 (≳ 50 μm)

Table S9: Refractive indices of agarose-sucrose samples calculated from measurements of primary spherical aberrations compared to the refractometer measurements.

Sample (shown in Fig. S6)	***A***	***B***	***C***
Concentration (m/v) of agarose (%)	0.8	1.98	2.37
Concentration (m/v) of sucrose (%)	33.4	31.8	68.0
n_3 *(refractometer at 656 nm)*	1.376	1.391	1.417
n_3 *(aberration measurements)*	1.375	1.393	1.417
95% confidence interval (from aberration measurements)	[1.364,1.386]	[1.393,1.396]	[1.412,1.423]

Table S10: Component name abbreviations used in the detailed setup overview in Fig. S1.

Component	Abbreviation
**Half-wave plate (HWP)**	$\lambda/2$
**Quarter-wave plate (QWP)**	$\lambda/4$
**DM**	Dichroic mirror
**PBS**	Polarizing beam splitter
**BS**	Beam splitter
**AOM**	Acousto-optic modulator
**AOTF**	Acousto-optic tunable filter
**PM fiber**	Polarization maintaining optical fiber (single mode)
**FC**	Fiber collimator/coupler
**TF**	Tunable filter
**APD**	Avalanche photodiode
**MM fiber**	Multimode optical fiber
**EOD**	Electro optical deflectors
**SLM**	Spatial light modulator (blazed grating to reflect into first order, phase masks)
**L**	Lens
**FPGA**	Field programmable gate array
**PH**	Pinhole
**PC**	Computer
**Flip DM**	Flip dichroic mirror (flip into beam path for widefield imaging)
**DMM**	Deformable membrane mirror
**FM**	Flip mirror
**F1**	Notch filters (Laser wavelengths, focus lock wavelengths)

Legends for Movies S1 to S3

Movie S1: Animation of Fig. 7*A*. The two-color 3D data of actin and PSD95 is rotated. Zoom-in to the post-synapse. Rotation of only PSD95 localizations.

Movie S2: Animation of Fig. 7*C*. The two-color 3D data of AMPAR and PSD95 is rotated. Zoom-in to the post-synapse and rotation of the post-synapse.

Movie S3: Animation of Fig. 7*D*. The two-color 3D data of actin and PSD95 is rotated. Zoom-in to two post-synapses and rotation of the PSD95 localizations.
