# Supplementary figures and images for: MINFLUX fluorescence nanoscopy in biological tissue
Source: Proc Natl Acad Sci U S A. 2024 Dec 20;121(52):e2422020121. doi: 10.1073/pnas.2422020121 (PMC11670107; doi:10.1073/pnas.2422020121)

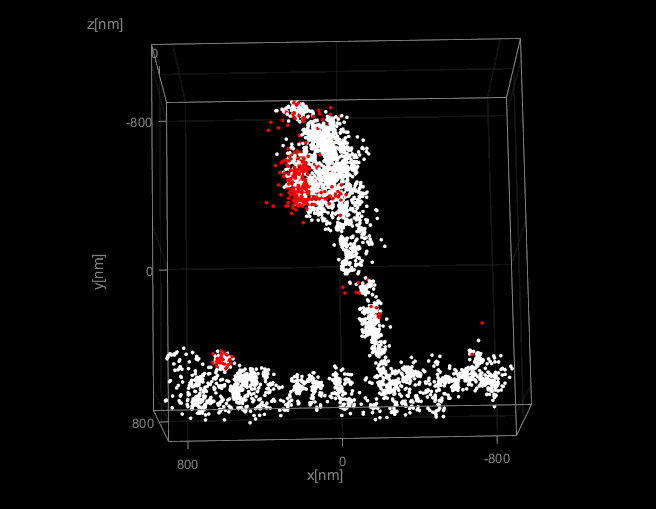

Supplement: Movie S1. — Animation of Fig. 7A. The two-color 3D data of actin and PSD95 is rotated. Zoom-in to the post-synapse. Rotation of only PSD95 localizations. [file pnas.2422020121.sm01.gif]

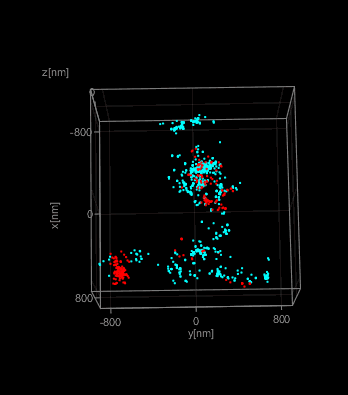

Supplement: Movie S2. — Animation of Fig. 7C. The two-color 3D data of AMPAR and PSD95 is rotated. Zoom-in to the post-synapse and rotation of the post-synapse. [file pnas.2422020121.sm02.gif]

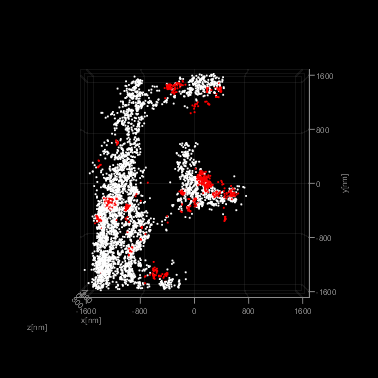

Supplement: Movie S3. — Animation of Fig. 7D. The two-color 3D data of actin and PSD95 is rotated. Zoom-in to two post-synapses and rotation of the PSD95 localizations. [file pnas.2422020121.sm03.gif]
